# Oxidative stress induces lysosomal membrane permeabilization and ceramide accumulation in retinal pigment epithelial cells

**DOI:** 10.1242/dmm.050066

**Published:** 2023-07-25

**Authors:** Kevin R. Zhang, Connor S. R. Jankowski, Rayna Marshall, Rohini Nair, Néstor Más Gómez, Ahab Alnemri, Yingrui Liu, Elizabeth Erler, Julia Ferrante, Ying Song, Brent A. Bell, Bailey H. Baumann, Jacob Sterling, Brandon Anderson, Sierra Foshe, Jennifer Roof, Hossein Fazelinia, Lynn A. Spruce, Jen-Zen Chuang, Ching-Hwa Sung, Anuradha Dhingra, Kathleen Boesze-Battaglia, Venkata R. M. Chavali, Joshua D. Rabinowitz, Claire H. Mitchell, Joshua L. Dunaief

**Affiliations:** ^1^ F.M. Kirby Center for Molecular Ophthalmology, Scheie Eye Institute, Perelman School of Medicine at the University of Pennsylvania, Philadelphia, PA 19104, USA; ^2^ Lewis-Sigler Institute for Integrative Genomics, Princeton University, Princeton, NJ 08544, USA; ^3^ Department of Molecular Biology, Princeton University, Princeton, NJ 08544, USA; ^4^ Department of Basic and Translational Sciences, School of Dental Medicine, University of Pennsylvania, Philadelphia, PA 19104, USA; ^5^ CHOP-PENN Proteomics Core Facility, The Children's Hospital of Philadelphia Research Institute, Philadelphia, PA 19104, USA; ^6^ Department of Ophthalmology, Weill Cornell Medicine, New York, NY 10065, USA; ^7^ Department of Cell and Developmental Biology, Weill Cornell Medicine, New York, NY 10065, USA

**Keywords:** Oxidative stress, Aging, Retina, Age-related macular degeneration, Lysosome

## Abstract

Oxidative stress has been implicated in the pathogenesis of age-related macular degeneration, the leading cause of blindness in older adults, with retinal pigment epithelium (RPE) cells playing a key role. To better understand the cytotoxic mechanisms underlying oxidative stress, we used cell culture and mouse models of iron overload, as iron can catalyze reactive oxygen species formation in the RPE. Iron-loading of cultured induced pluripotent stem cell-derived RPE cells increased lysosomal abundance, impaired proteolysis and reduced the activity of a subset of lysosomal enzymes, including lysosomal acid lipase (LIPA) and acid sphingomyelinase (SMPD1). In a liver-specific *Hepc* (*Hamp*) knockout murine model of systemic iron overload, RPE cells accumulated lipid peroxidation adducts and lysosomes, developed progressive hypertrophy and underwent cell death. Proteomic and lipidomic analyses revealed accumulation of lysosomal proteins, ceramide biosynthetic enzymes and ceramides. The proteolytic enzyme cathepsin D (CTSD) had impaired maturation. A large proportion of lysosomes were galectin-3 (Lgals3) positive, suggesting cytotoxic lysosomal membrane permeabilization. Collectively, these results demonstrate that iron overload induces lysosomal accumulation and impairs lysosomal function, likely due to iron-induced lipid peroxides that can inhibit lysosomal enzymes.

## Teaser

**Summary:** Oxidative stress from iron loading in retinal pigment epithelial cells induces lysosomal accumulation and impairs lysosomal function, leading to lysosomal membrane permeabilization, ceramide accumulation and cell death.

## INTRODUCTION

Oxidative stress has been implicated in the pathogenesis of age-related macular degeneration (AMD). Antioxidant vitamins reduce the risk of AMD progression in clinical trials (Age-Related Eye Disease Study Research Group, 2001). Oxidized lipids, adducts bound to lipid peroxidation products, advanced glycation end products and iron have all been found at elevated levels in postmortem AMD maculas relative to age-matched controls (Gu et al., 2003; Hahn et al., 2003; Suzuki et al., 2007; Yamada et al., 2006). Yet, understanding of the mechanisms by which oxidative stress promotes AMD and the role of these factors in its pathogenesis remains incomplete.

Iron is vital in numerous biochemical processes owing to its role as a cofactor of many enzymes (Beard, 2001). However, iron can also catalyze the production of reactive oxygen species (ROS) through the Fenton reaction (Jankowski and Rabinowitz, 2022; Winterbourn, 1995). Iron-dependent ROS formation causes lipid peroxidation and the production of the toxic molecules 4-hydroxynonenal (4-HNE), malondialdehyde and carboxyethylpyrrole (CEP). These lipid peroxidation products can impair enzyme function, alter membrane fluidity and disrupt intermolecular interactions (Lewandowski et al., 2022). Iron-catalyzed ROS and lipid peroxidation damages numerous tissues, with iron overload implicated in diseases ranging from diabetes and atherosclerosis to neurodegenerative diseases, including Alzheimer's disease and Parkinson's disease (de Valk and Marx, 1999; Liu et al., 2018; Mochizuki et al., 2020; Simcox and McClain, 2013). Iron has a particularly strong association with age-related diseases, given its tendency to accumulate over time and the lack of specialized organismal-level iron-excretion systems (Chen et al., 2022).

The retina is especially vulnerable to iron-catalyzed ROS owing to its high metabolic rate, high oxygen tension and exposure to photo-oxidizing light (Rogers et al., 2007). Consistent with these features of retinal physiology, substantial evidence exists correlating iron overload to retinal diseases including AMD, the leading cause of blindness in individuals over the age of 50 years (Hahn et al., 2003). Patients with AMD have elevated levels of iron in multiple layers of the retina, including a layer of supportive cells in the outer retina known as the retinal pigment epithelium (RPE) (Hahn et al., 2003). The early onset of AMD-like pathology following intravenous iron supplementation (Song et al., 2016) and early-onset macular degeneration in a patient with retinal and RPE iron overload due to aceruloplasminemia have also been reported (Dunaief et al., 2005; Wolkow et al., 2011). This mechanism of oxidative damage in the retina appears to be conserved, as several mouse models with elevated retinal iron have also been found to develop an AMD-like pathology (Hahn et al., 2004; Liu et al., 2021). However, the mechanisms by which iron and, more broadly, oxidative stress induce retinal damage are unclear.

Degeneration of the RPE plays a major role in the pathogenesis of AMD. The RPE supplies photoreceptors with glucose, forms a blood-retinal barrier and maintains photoreceptor health by phagocytosing photoreceptor outer segments (Chao et al., 2017; Kanow et al., 2017). Nearly 10% of photoreceptor outer segment volume is shed daily and subsequently phagocytosed by the adjacent RPE (Kevany and Palczewski, 2010; Reyes-Reveles et al., 2017). This high rate of phagocytosis is demanding on RPE lysosomes, which must digest and clear the phagocytosed material. Incomplete lysosomal digestion leads to the formation of intracellular lipofuscin, a mixture of autofluorescent lipids and proteins that accumulate with age in humans (Ach et al., 2014) and have been shown in mice to be toxic to RPE cells (Pan et al., 2021). Lysosomal dysfunction has been implicated in AMD, as RPE cells from patients with AMD have enlarged organelles positive for the lysosomal membrane marker Lamp1 (Golestaneh et al., 2017). Furthermore, the addition of the bisretinoid N-retinyl-N-retinylidene ethanolamine (A2E), a major component of non-macular lipofuscin, to cultured RPE cells increases lysosomal pH (Liu et al., 2008).

To examine the impact of iron-induced oxidative stress on RPE lysosomal function, we triggered oxidative stress in human induced pluripotent stem cell-derived-RPE (iPS-RPE) cells with chronic iron overload. This causes lysosomal accumulation, inhibits lysosomal proteolysis and inhibits the activity of several lysosomal enzymes. We also tested the *in vivo* effects of iron on RPE lysosomal function using a liver-specific hepcidin (*Hepc*, also known as *Hamp*) knockout (LS-*Hepc*^KO^; genotype: AlbCre^+^, *Hepc*^f/f^) mouse model previously developed in our laboratory (Baumann et al., 2019). Hepcidin is a regulatory hormone primarily synthesized by hepatocytes that downregulates expression of ferroportin (Fpn, encoded by *Slc40a1*), the only known mammalian iron exporter. Hepcidin binds to ferroportin, leading to its internalization from the cell membrane and subsequent degradation. Loss of hepcidin allows intestinal enterocytes to constantly transport iron into the bloodstream, leading to elevated blood iron levels (Hadziahmetovic et al., 2011). The LS-*Hepc*^KO^ model develops elevated serum iron levels and accumulation of iron in the RPE and neural retina (Baumann et al., 2019). The RPE thickens with cells piling on top of each other, although the lack of mitotic cells suggests that this is due to hypertrophy and not hyperplasia. RPE cells also become autofluorescent and accumulate numerous lipofuscin-filled vesicles, although the identity of these vesicles was undefined (Baumann et al., 2019).

In the present study, we explored the mechanisms explaining the development of pathology in the iron-loaded RPE of LS-*Hepc*^KO^ mice and correlated the phenotypes to those seen in AMD. We observed many vesicular structures by electron microscopy (EM) and identified them as lysosomes. We provide evidence of lysosomal dysfunction and lysosomal membrane permeabilization (LMP), and demonstrate that the autofluorescent material is intralysosomal. We further show that LS-*Hepc*^KO^ lysosomes accumulated lipid peroxidation products and numerous other lipid species, including ceramides and lysophosphatidylcholines (LPCs). The ultimate result of the lysosomal dysfunction and lipid accumulation was cell death, contributing to many RPE phenotypes also seen in AMD. Taken together, our *in vitro* and *in vivo* results suggest that oxidative stress may contribute to the pathology of AMD by disrupting lysosomal function and subsequently impairing the lysosomal clearance of autophagy cargo and phagocytosed outer segments.

## RESULTS

### Iron-loaded iPS-RPE cells accumulate lysosomes with impaired enzyme activity

Human iPS-RPE cells were used as an *in vitro* model. Characterization of these cells was carried out in cultures grown for a minimum of 4 weeks after appearance of RPE-like cobblestone morphology and used for experiments thereafter. iPS-RPE cells expressed the tight junction protein ZO-1 (or TJP1) and RPE65 (Fig. S1A), displayed a transepithelial electrical resistance of 327.8±5.32 Ω cm^2^ (mean±s.e.m.) after 4 weeks in culture (Fig. S1B), and expressed RPE-specific markers, including *BEST1*, *MITF*, *RLBP1* and *RPE65* (Fig. S1C). Genotyping for single-nucleotide polymorphisms associated with AMD was performed on these cells and demonstrated heterozygosity for the risk allele in both *ARMS2* (rs10490924: G;T; Rivera et al., 2005) and *CFH* (rs1061170: C;T; Despriet et al., 2006), both resulting in an elevated risk of AMD of 2.5×.

**Fig. 1.**
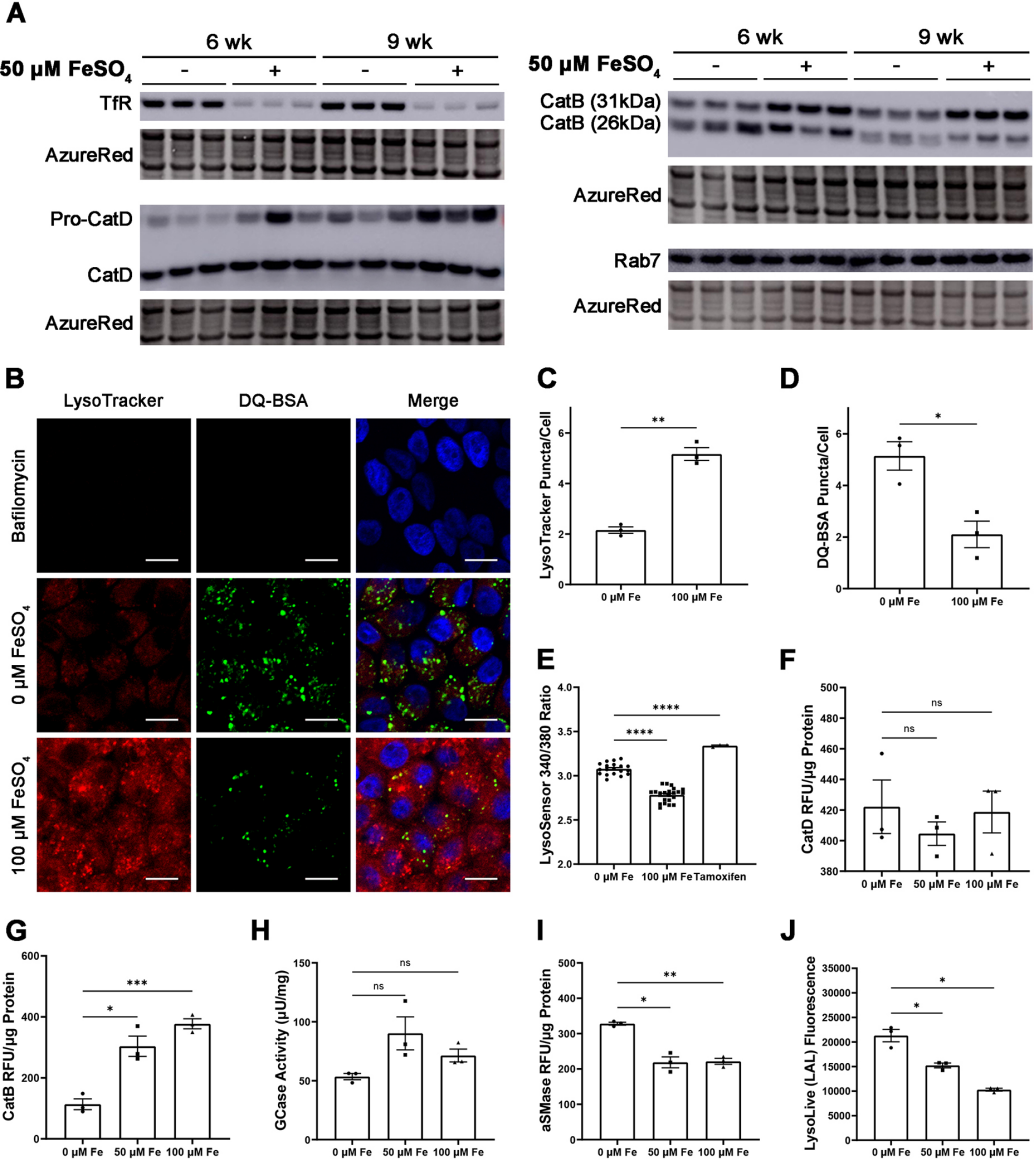
**Iron-loaded iPS-RPE accumulate lysosomes with impaired enzyme activity.** (A) Western blot analysis of human iPS-RPE loaded with 50 μM FeSO_4_ for 6 and 9 weeks. AzureRed total protein stain served as a loading control. Each lane represents one experimental replicate. (B) Representative confocal images of human iPS-RPE cells loaded with LysoTracker (red) to detect lysosomes and DQ-BSA (green) to detect lysosomal proteolysis. Nuclei are labeled with DAPI (blue). Scale bars: 10 μm. (C) Quantification of LysoTracker-positive puncta per cell. (D) Quantification of DQ-BSA-positive puncta per cell. (E) Measurement of lysosomal pH using LysoSensor Yellow/Blue, with higher values of the 340/380 ratio correlating to increased pH. (F-J) Enzyme activity assays for cathepsin D (F), cathepsin B (G), glucosylceramidase (GCase) (H), acid sphingomyelinase (aSMase) (I) and lysosomal acid lipase (LAL) (J). Cells were incubated with 0, 50 or 100 μM FeSO_4_ for 6 weeks in B-J. Values in graphs represent the mean±s.e.m. RFU, relative fluorescence units. ns, not significant; *P<0.05; **P<0.01; ***P<0.001; ****P<0.0001 (two-tailed unpaired Student's *t*-test).

To determine whether iron affects lysosomes in RPE cells, we cultured these iPS-RPE cells with FeSO_4_ for 6 or 9 weeks. This chronic iron exposure caused elevated intracellular iron levels, as indicated by diminished levels of the iron-regulated transferrin receptor (TfR or TFRC) protein (Fig. 1A; Fig. S2). Cathepsin D (CatD or CTSD), a lysosomal aspartic protease, migrated as two bands with apparent masses of 48 and 34 kDa. The 48 kDa band corresponds to the immature form, pro-cathepsin D, which undergoes proteolytic cleavage by cathepsin B or L in the lysosome to generate a mature two-chain enzyme consisting of a heavy 34 kDa and light 14 kDa chain (Zaidi et al., 2008). Pro-cathepsin D levels were increased by iron supplementation at both 6 and 9 weeks, although a statistically significant difference was only apparent after 9 weeks. Mature cathepsin D levels were not significantly different at either timepoints (Fig. 1A; Fig. S2).

Western blot analysis of cathepsin B (CatB or CTSB) in iron-loaded iPS-RPE cells demonstrated two bands with masses of approximately 31 kDa and 26 kDa (Fig. 1A). Iron loading for 9 weeks significantly increased the levels of the 31 kDa form, which is produced in endosomes (Cavallo-Medved et al., 2011). The levels of the 26 kDa chain produced in the lysosomes were not changed by iron. The levels of Rab7 (or RAB7A), a marker frequently associated with late endosomes (Humphries et al., 2011; Shearer and Petersen, 2019), were not changed by iron at 6 weeks and only mildly increased at 9 weeks (Fig. 1A; Fig. S2). Although Rab7 can also be found on lysosomes, the increase in cathepsin levels with minimal change in Rab7 levels suggests a lysosome-specific phenotype caused by iron.

To determine whether iron affects the number of lysosomes per cell and lysosomal proteolysis, iPS-RPE cells were loaded with iron for 6 weeks, and then exposed to LysoTracker to visualize acidic organelles and DQ-BSA Green to measure lysosomal proteolytic function (Fig. 1B). DQ-BSA becomes fluorescent following proteolytic cleavage in a pH-independent manner. Bafilomycin, a lysosomal v-ATPase inhibitor that neutralizes luminal pH (Wang et al., 2021), eliminated LysoTracker and DQ-BSA fluorescence as expected. Cells loaded with FeSO_4_ displayed a significant increase in LysoTracker-positive puncta on a per-cell basis (Fig. 1B,C), along with a significant decrease in DQ-BSA positive puncta (Fig. 1B,D). These data suggest that although iron induces lysosomal accumulation, many of these lysosomes appear to be dysfunctional. Given the significant results at this time point, additional lysosomal characterization experiments were performed on cells incubated with FeSO_4_ for 6 weeks to correlate results to the LysoTracker and DQ-BSA study.

As the function of many lysosomal enzymes requires an acidic pH, a proteolysis deficit could reflect lysosomal deacidification. We used LysoSensor Yellow/Blue to measure lysosomal pH in iPS-RPE cells loaded with iron for 6 weeks. LysoSensor Yellow/Blue is a ratiometric dye, with a higher ratio of fluorescence signal at 340 nm versus that at 380 nm (hereafter 340/380 ratio), indicative of higher pH (Guha et al., 2014). We unexpectedly found a lower 340/380 ratio with iron, suggesting that iron incubation decreased the overall lysosomal pH in these cells (Fig. 1E). Based on the magnitude of the decrease, we did not expect this lower pH to significantly impact cellular physiology or activity of lysosomal enzymes. Tamoxifen, which increases lysosomal pH (Altan et al., 1999), significantly increased the 340/380 ratio as expected. These results suggested that the proteolysis deficit indicated by DQ-BSA was not a result of lysosomal deacidification.

Given a generalized proteolysis deficit without concomitant impairment in lysosomal acidification, we measured the activity of lysosomal cathepsins to determine whether cathepsin dysfunction may explain the decreased proteolysis. Although western blot analysis indicated increased levels of pro-cathepsin D (Fig. 1A), we did not observe any iron-induced change in cathepsin D activity at 6 weeks (Fig. 1F). Interestingly, cathepsin B activity was significantly increased by iron at 6 weeks (Fig. 1G). This may reflect increased levels of the 31 kDa form of cathepsin B, as both the 31 kDa and 26 kDa forms of cathepsin B are enzymatically active (Cavallo-Medved et al., 2011; Mort and Buttle, 1997). The activity of three other lysosomal enzymes were differentially affected by iron at 6 weeks. Glucosylceramidase (GCase or GBA) activity was unchanged by iron (Fig. 1H), whereas the activities of acidic sphingomyelinase (aSMase or SMPD1) (Fig. 1I) and lysosomal acid lipase (LAL or LIPA) (Fig. 1J) were both decreased by iron.

### The LS-*Hepc*^KO^ RPE is hypertrophic and autofluorescent

**Fig. 2.**
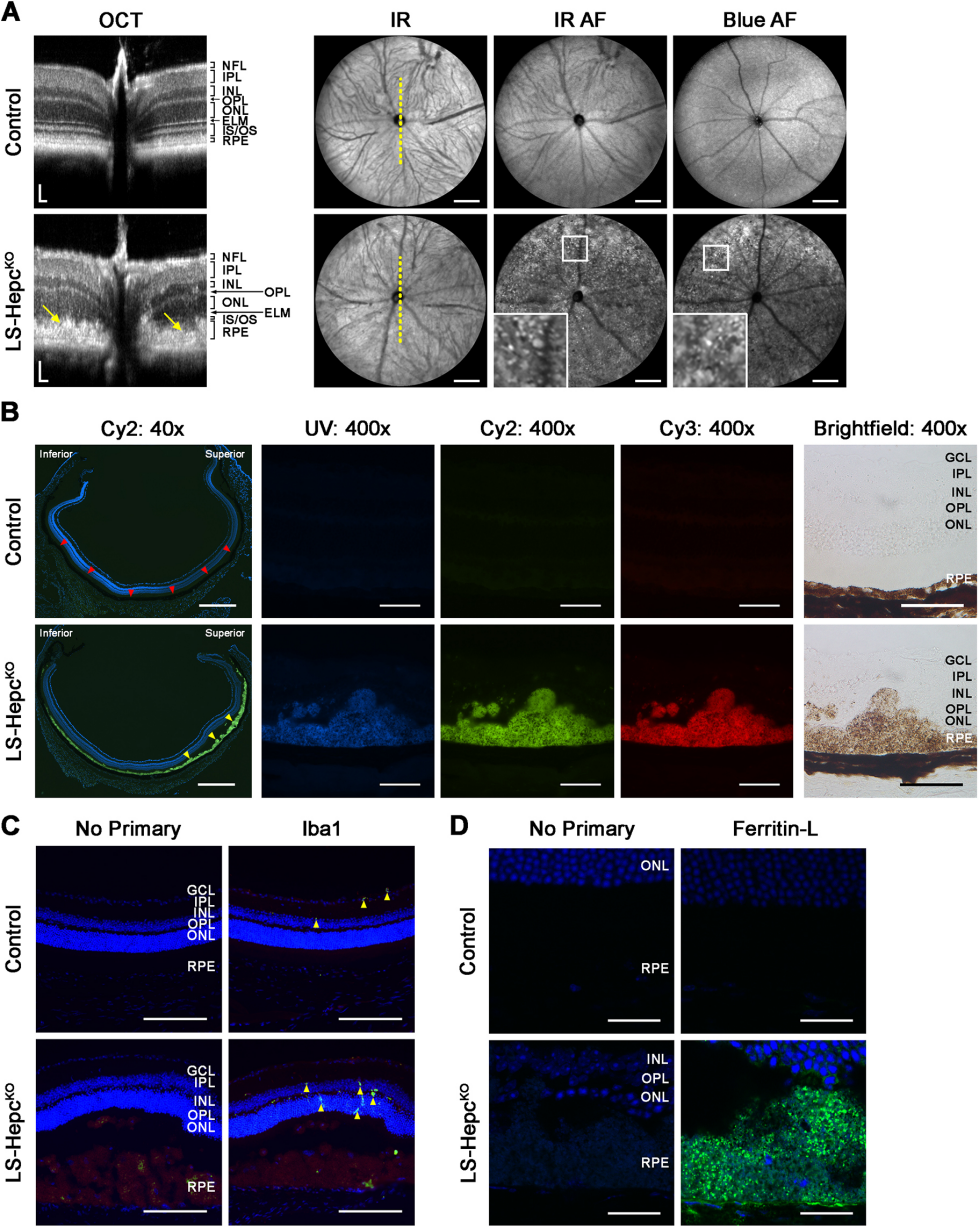
**LS-*Hepc*^KO^ RPE is hypertrophic and autofluorescent.** (A) Representative optical coherence tomography (OCT) (left) and confocal scanning laser ophthalmoscopy (cSLO) (right) images from 12-month-old LS-*Hepc*^KO^ (*n*=4) and control (*n*=4) mice. OCT scans were taken vertically through the optic nerve, as indicated by the dotted yellow line, with a field of view about half that of the cSLO. Arrows on the OCT images point to hypertrophic RPE. Regions bounded by white squares are enlarged in the inset and display autofluorescent foci. IR, infrared; AF, autofluorescence. Scale bars: 50 μm (horizontally and vertically, OCT); 500 μm (cSLO). (B) Autofluorescence and brightfield imaging of cryosections. Red arrowheads indicate the RPE in the control; yellow arrowheads indicate regions of RPE hypertrophy in LS-*Hepc*^KO^ sections. Scale bars: 500 μm (40× images); 50 μm (400× images). (C) Immunofluorescence for Iba1 (green) in 12-month-old mice. Yellow arrowheads point to Iba1^+^ cells. Scale bars: 200 μm. (D) Representative immunofluorescence confocal images for ferritin-L (green) in 12-month-old mice. Scale bars: 25 μm. Nuclei are labeled with DAPI (blue) in the 40× images of B, as well as in C,D. Control mice had the genotype AlbCre^−^, *Hepc*^f/f^ in A and were wild type in B-D. Experiments without any primary antibody (‘no primary’) were performed as controls. Representative immunolabeling images are shown from *n*=4 mice per genotype in B-D. ELM, external limiting membrane; GCL, ganglion cell layer; INL, inner nuclear layer; IPL, inner plexiform layer; IS, inner segments; NFL, nerve fiber layer; ONL, outer nuclear layer; OPL, outer plexiform layer; OS, outer segments; RPE, retinal pigment epithelium.

To determine whether the iron-induced lysosomal accumulation and enzyme inhibition observed in iPS-RPE cells would also occur *in vivo*, we examined the LS-*Hepc*^KO^ mouse model previously developed in our laboratory (Baumann et al., 2019), which develops progressive retinal and RPE iron overload. Pathology in this model begins with sparse RPE hypertrophy (enlargement both horizontally and vertically, along with rounding) at 6 months, progressing to near global RPE hypertrophy by 12 months. We began by verifying the previously observed phenotype and extended the characterization of the model using *in vivo* imaging in 12-month-old mice. Control mice had the genotype AlbCre^−^, *Hepc*^f/f^. Optical coherence tomography (OCT) scans taken along the vertical meridian (Fig. 2A) demonstrated broadening of the hyperreflective band at the level of the RPE (yellow arrows), consistent with RPE hypertrophy, and marked thinning of the inner and outer segment layers as previously described (Baumann et al., 2019). Confocal scanning laser ophthalmoscopy (cSLO) showed numerous autofluorescent foci in both near-infrared and short-wavelength (blue) autofluorescence images. Microscopy of cryosections from 12-month-old LS-*Hepc*^KO^ mouse retinas demonstrated RPE autofluorescence with patches of massive RPE hypertrophy (Fig. 2B). The hypertrophy was more severe in the superior retina (arrowheads). This contrasts to the uniform, thinner, non-autofluorescent RPE of age-matched wild-type controls. The hypertrophic cells also displayed strong autofluorescence in the ultraviolet (UV), Cy2 and Cy3 channels, with clumps of these autofluorescent RPE cells likely corresponding to the autofluorescent foci noted earlier by cSLO (Fig. 2A).

Owing to the drastically altered appearance of the RPE, we sought to confirm that these hypertrophic cells represented bona fide RPE cells. Pathologic RPE often de-differentiates or transdifferentiates, making it difficult to use traditional markers of RPE including RLBP1, RPE65 or BEST1 (Alge et al., 2003; Cao et al., 2021; Plaza Reyes et al., 2020). Given the gradual, age-dependent increase in size of these initially monolayered autofluorescent cells and their position on Bruch's membrane, their identity was most consistent with that of RPE. However, it was possible that the hypertrophic cells were infiltrating macrophages and microglia that entered the subretinal space to phagocytose dying RPE. Immunohistochemistry (IHC) for Iba1 (or Aif1), a marker of microglia and macrophages, demonstrated Iba1-positive cells throughout the neural retina of both LS-*Hepc*^KO^ and age-matched wild-type mice (arrowheads), as expected, whereas the hypertrophic cells were Iba1 negative (Fig. 2C). Occasional dim fluorescent signals were noticed in the hypertrophic cells, but this reflects RPE autofluorescence rather than true Iba1 positivity given the dimness and pattern of the signal and the presence of this fluorescence in the no-primary-antibody control.

To assess the extent of iron accumulation in the LS-*Hepc*^KO^ RPE, we examined the retinal distribution of ferritin, the primary iron storage protein (Mackenzie et al., 2008), by IHC. Confocal microscopy demonstrated strong ferritin-L (Ftl1) immunolabeling in 12-month LS-*Hepc*^KO^ RPE, confirming iron overload (Fig. 2D), whereas comparatively little immunolabeling was observed in age-matched wild-type controls.

### The LS-*Hepc*^KO^ RPE accumulates lysosomes

**Fig. 3.**
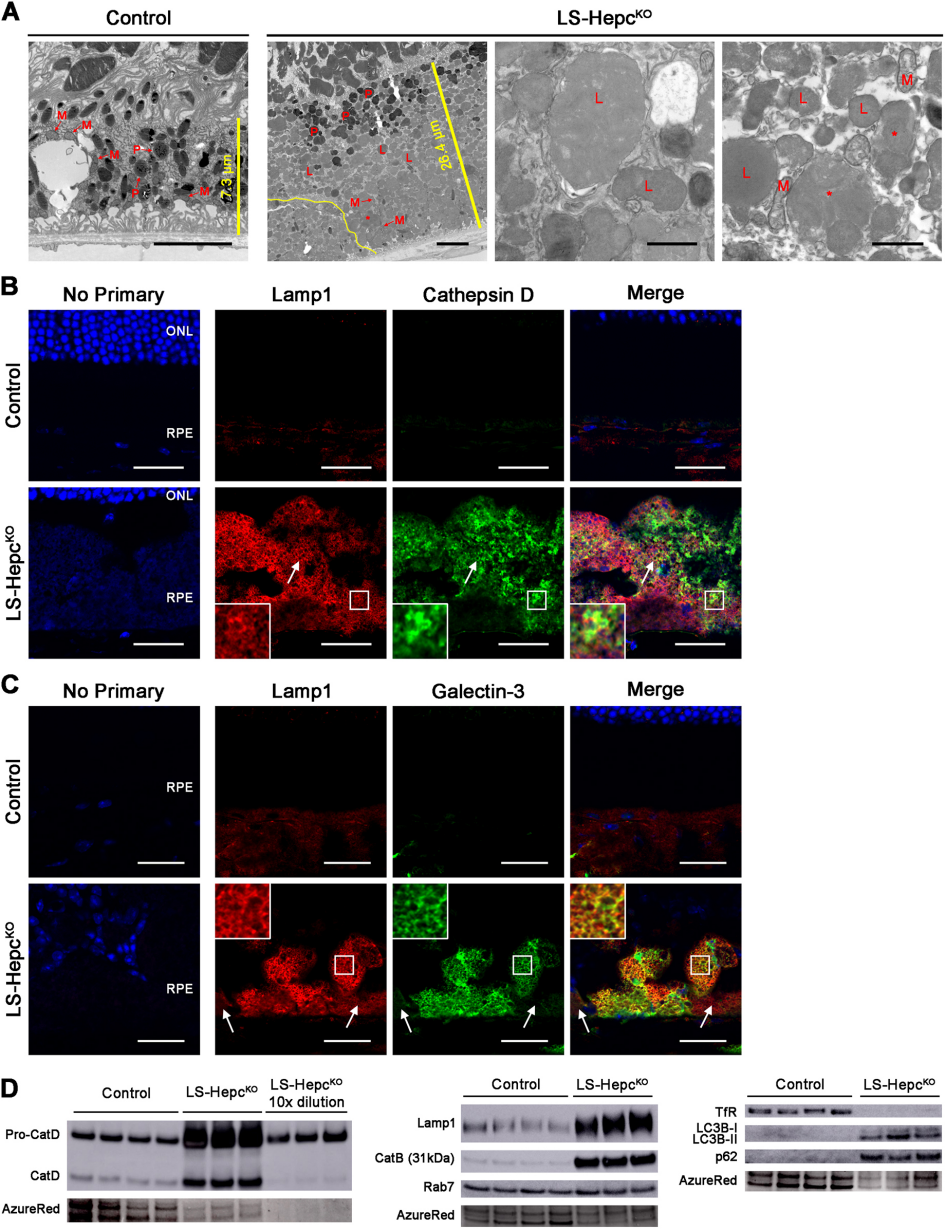
**LS-*Hepc*^KO^ RPE accumulate lysosomes.** (A) Electron micrographs of RPE from 12-month-old mice (*n*=4). The first two images are lower magnification and span the height of one RPE cell; scale bars: 5 μm. The last two images are higher magnification; scale bars: 1 μm. L, lysosome; M, mitochondrion; P, phagolysosome; *, multivesicular body. The straight yellow lines in the first two images indicate the height of the RPE cell, with lengths indicated. The non-linear yellow line in the second image outlines the border between two RPE cells. (B) Confocal images of immunofluorescence for Lamp1 (red) and cathepsin D (green) in 12-month-old mice. White arrows indicate an example of Lamp1 and cathepsin D colocalization. (C) Confocal images of Lamp1 (red) and galectin-3 (green) immunofluorescence in 12-month-old mice. White arrows show flanking cells without significant galectin-3 expression. Regions bounded by white squares in B,C are enlarged in the insets. Nuclei are labeled with DAPI (blue). Experiments without any primary antibody (‘no primary’) were performed as controls. Representative immunolabeling images are shown from *n*=4 mice per genotype. Control mice had the genotype AlbCre^−^, *Hepc*^f/f^ in A and D and were wild type in B,C. ONL, outer nuclear layer; RPE, retinal pigment epithelium. Scale bars: 25 μm. (D) Western blot analysis on isolated RPEs from 12-month-old mice. AzureRed total protein stain served as a loading control. Each lane represents protein from one mouse.

To more closely examine the structure of the hypertrophic RPE cells in LS-*Hepc*^KO^ mice, we used EM to image RPE cells in 12-month-old mice (Fig. 3A). Counterstaining was performed using the osmium tetroxide-thiocarbohydrazide-osmium (OTO) method, which preferentially labels lipid-rich structures (Seligman et al., 1966). The heights of individual RPE cells of LS-*Hepc*^KO^ were three to four times the height of the RPE cells of age-matched controls, and LS-*Hepc*^KO^ RPE cells occasionally also piled on top of each other (yellow line denotes the border between two cells), in contrast to the monolayer of cells formed by healthy RPE. These morphologies have been observed in the RPE of patients with AMD (Zanzottera et al., 2015). Control RPE had apical microvilli and abundant melanosomes, mitochondria and phagolysosomes. LS-*Hepc*^KO^ RPE cells contained phagolysosomes; however, they had sparse melanosomes and mitochondria, and were instead packed with electron-dense inclusions. Higher-magnification images showed that these inclusions were bound by a single membrane and filled with a homogeneous, moderately electron-dense substance. Some of these inclusions appeared to be multivesicular bodies (Fig. 3A, red asterisks).

We sought to determine whether these inclusions were derived from lipid-laden lysosomes by using IHC for the lysosomal membrane marker Lamp1 and cathepsin D (Fig. 3B). Confocal microscopy demonstrated Lamp1-positive structures throughout the RPE of 12-month-old LS-*Hepc*^KO^ mice, supporting a lysosomal identity of the inclusions. Although Lamp1 and cathepsin D were co-labeled in some areas (white arrow), Lamp1 tended to mark membrane structures, whereas cathepsin D was often detected in the cytoplasmic spaces between the Lamp1-labeled structures. This suggested that the lysosomes of LS-*Hepc*^KO^ RPE may be damaged, with features of LMP demonstrated by cathepsin D leaking into the surrounding cytoplasm.

To determine whether LMP may be occurring in the RPE of 12-month-old LS-*Hepc*^KO^ mice, we co-labeled galectin-3 (or Lgals3) and Lamp1. When lysosomal membranes become damaged and leaky, galectin-3 translocates from the cytoplasm to the damaged lysosomal membrane, thus serving as a sensitive method of assessing LMP (Aits et al., 2015). Many individual RPE cells of LS-*Hepc*^KO^ contained patchy heterogeneous immunolabeling for galectin-3 (Fig. 3C). The RPE layer as a whole displayed mosaic galectin-3 immunolabeling, with some cells displaying strong, uniform galectin-3 immunolabeling juxtaposed to cells without galectin-3 immunolabeling (white arrows point to two cells without galectin-3 immunolabeling that flank cells with strong immunolabeling). The galectin-3 immunolabeling colocalized with Lamp1 immunolabeling in almost all locations. Galectin-3 immunolabeling was particularly strong in cells that appeared round and detached from Bruch's membrane (on the basal side of normal RPE), whereas cells that were less hypertrophic and attached to Bruch's membrane were frequently negative for galectin-3 immunoreactivity. This morphology is reminiscent of pathologic RPE cells at the edge of geographic atrophy in AMD eyes (Zanzottera et al., 2015). These data suggest that in older LS-*Hepc*^KO^ mice, lysosomes accumulate and become leaky, corresponding to RPE cell migration and de-differentiation.

Iron overload and lysosomal function in LS-*Hepc*^KO^ RPE was further characterized using western blot analysis of isolated RPE cells from 12-month-old knockout and control mice. The protein levels of TfR were almost undetectable in LS-*Hepc*^KO^ RPE, indicative of iron overload (Fig. 3D). LS-*Hepc*^KO^ RPE displayed much higher levels of both pro-cathepsin D and mature cathepsin D compared to those of age-matched controls, consistent with increased lysosome number – the signal from pro-cathepsin D was greatly oversaturated, so samples diluted tenfold were also tested. The ratio of pro-cathepsin D to mature cathepsin D was six times higher in the LS-*Hepc*^KO^ RPE compared to that in the control RPE, suggesting a defect in lysosomal cathepsin D proteolysis (Fig. S3).

The protein levels of both Lamp1 and cathepsin B were also markedly increased in the LS-*Hepc*^KO^ RPE (Fig. 3D). Compared to their levels in controls, Lamp1 was about four times more abundant and cathepsin B was 17 times more abundant in LS-*Hepc*^KO^ RPE (Fig. S3). The levels of Rab7 were not significantly different between the LS-*Hepc*^KO^ and control RPEs. These data together provided strong evidence that lysosomes specifically accumulated in the LS-*Hepc*^KO^ RPE. Because damaged lysosomes can be cleared by lysophagy, the selective autophagy of lysosomes, we measured the levels of LC3B-II (encoded by *Map1lc3b*), which localizes to autophagosome membranes, and p62 (encoded by *Sqstm1*), the autophagic substrate (Klionsky et al., 2021). Both LC3B-II and p62 were markedly increased in LS-*Hepc*^KO^ RPE (Fig. 3D), with LC3B-II and p62 levels both about four times those of controls (Fig. S3). This suggests an accumulation of autophagosomes in LS-*Hepc*^KO^ RPE, possibly owing to impaired autophagosome turnover.

### Proteomics reveals enrichment of lysosomal proteins in LS-*Hepc*^KO^ RPE

**Fig. 4.**
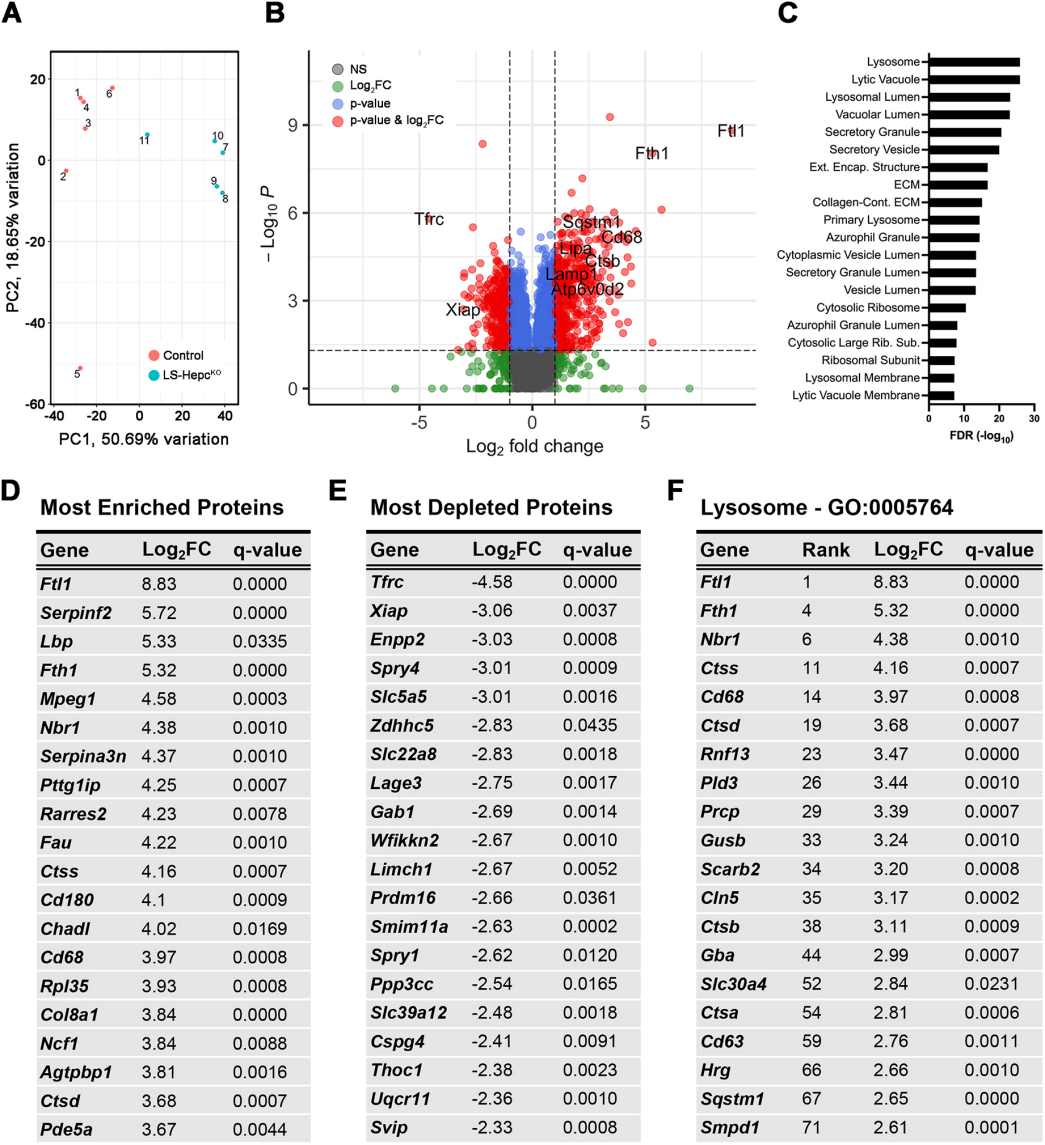
**Proteomics reveals enrichment of lysosomal proteins in LS-*Hepc*^KO^ RPE.** (A) Principal component analysis for RPE isolated from 12-month-old LS-*Hepc*^KO^ and control AlbCre^−^, *Hepc*^f/f^ mice. (B) Volcano plot for proteins enriched in LS-*Hepc*^KO^ versus control RPEs. Colored circles represent proteins that met criteria for adjusted *P*-value<0.05 and/or absolute value of log_2_ fold change (|log_2_FC|)>1. (C) Enrichment analysis of cellular components with the ToppGene Suite for LS-*Hepc*^KO^ versus control RPEs. Categories correspond to Gene Ontology (GO) terms. (D) Table of the 20 most enriched proteins in 12-month-old LS-*Hepc*^KO^ RPE. (E) Table of the 20 most depleted proteins in 12-month-old LS-*Hepc*^KO^ RPE. (F) Table of the 20 most enriched lysosomal proteins in 12-month-old LS-*Hepc*^KO^ RPE, based on annotation in GO:0005764. Rank denotes their overall placement by log_2_FC amongst all identified proteins.

We performed proteomics analysis on the RPE from 12-month-old LS-*Hepc*^KO^ and age-matched AlbCre^−^, *Hepc*^f/f^ control mice to better understand the molecular changes underlying the RPE hypertrophy and autofluorescence. A total of 5758 proteins with a minimum of three non-missing values in at least one of the genotype groups were identified. Of these, 2500 proteins were differentially expressed with an adjusted *P*-value of <0.05, and 814 proteins additionally met the criteria of having the absolute value of log_2_(fold change or FC)>1. Principal component analysis demonstrated separation between LS-*Hepc*^KO^ and control (Fig. 4A). Positive values for the log_2_FC indicate greater levels in LS-*Hepc*^KO^ compared to those in control. Consistent with expectations for iron-loaded cells, TfR was the most depleted protein in LS-*Hepc*^KO^, and the iron storage proteins ferritin-L (Ftl1) and ferritin-H (Fth1) were the most and fourth-most enriched proteins, respectively (Fig. 4B). Several proteins traditionally used as RPE markers were diminished in LS-*Hepc*^KO^, such as Rpe65 (log_2_FC=−1.10), Rlbp1 (log_2_FC=−1.57) and Lrat (log_2_FC=−2.16), suggesting RPE de-differentiation (Table S2).

Gene Ontology analysis using the ToppGene Suite (Chen et al., 2009b) was performed on proteins with log_2_FC>1 (Fig. 4C). This revealed enrichment of lysosomal proteins, in concordance with aforementioned data indicating lysosomal accumulation (Fig. 3). We also performed additional enrichment analysis in the context of human phenotype and disease (Fig. S4). Interestingly, AMD was listed among the associated diseases. Lysosomal proteins were strongly enriched in the LS-*Hepc*^KO^ RPE, with six of the top 20 enriched proteins falling under the lysosome term (GO:0005764) (Fig. 4D,F). Although one of these, CD63, is traditionally used as a marker of myelocytes, it is also expressed by RPE cells (Wang et al., 2009). Mitochondrial proteins (GO:0005739) were overall depleted in LS-*Hepc*^KO^, such as Spry4 (log_2_FC=−3.01), Ppp3cc (log_2_FC=−2.54) and Uqcr11 (log_2_FC=−2.36) (Fig. 4E).

### LS-*Hepc*^KO^ RPE lysosomes accumulate autofluorescent material and neutral lipids

**Fig. 5.**
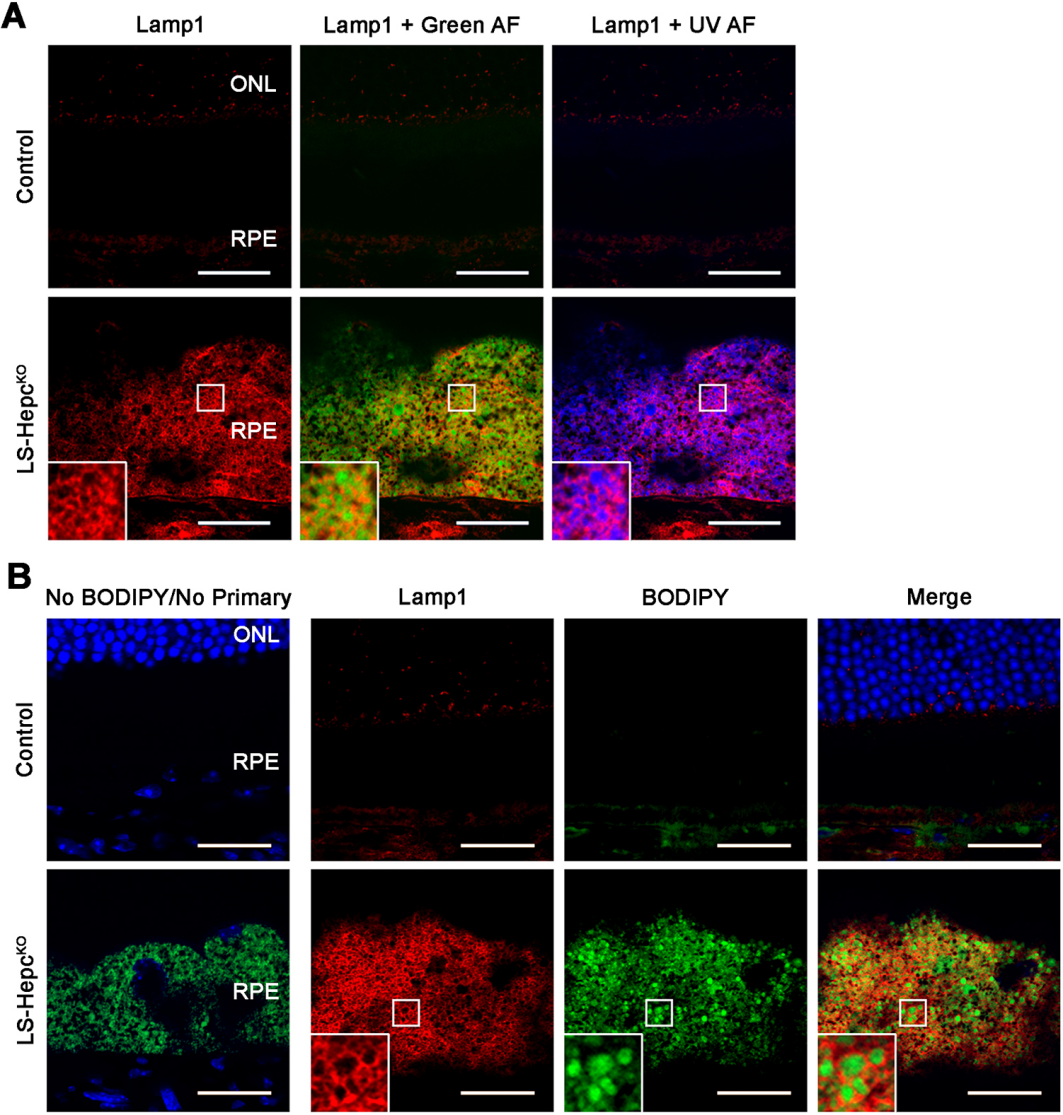
**LS-*Hepc*^KO^ RPE lysosomes accumulate autofluorescent material and neutral lipids.** (A) Representative confocal images of autofluorescence (AF) and Lamp1 immunofluorescence (red) in 12-month-old mice. (B) Confocal images of BODIPY 493/503 (for neutral lipids) fluorescence (green) and Lamp1 immunofluorescence (red) in 12-month-old mice. Nuclei are labeled with DAPI (blue). Regions bounded by white squares are enlarged in insets. Control mice were wild type. Representative immunolabeling images are shown from *n*=4 mice per genotype. ONL, outer nuclear layer; RPE, retinal pigment epithelium. Scale bars: 25 μm.

Having established that the LS-*Hepc*^KO^ RPE accumulates lysosomes, we sought to characterize the composition of their contents. Confocal fluorescence microscopy of Lamp1 and native RPE autofluorescence revealed that the autofluorescent compounds in LS-*Hepc*^KO^ RPE were intralysosomal (Fig. 5A). As EM with a lipid-enhancing technique demonstrated a moderately electron-dense material within the lysosomes (Fig. 3A), we hypothesized that lipids were a major contributor to the autofluorescence. Fluorescence from BODIPY 493/503, a neutral lipid stain, was observed within Lamp1-positive vesicles, confirming intralysosomal lipid accumulation (Fig. 5B).

### The LS-*Hepc*^KO^ RPE accumulates ceramides, acyl carnitines and docosahexaenoic acid

**Fig. 6.**
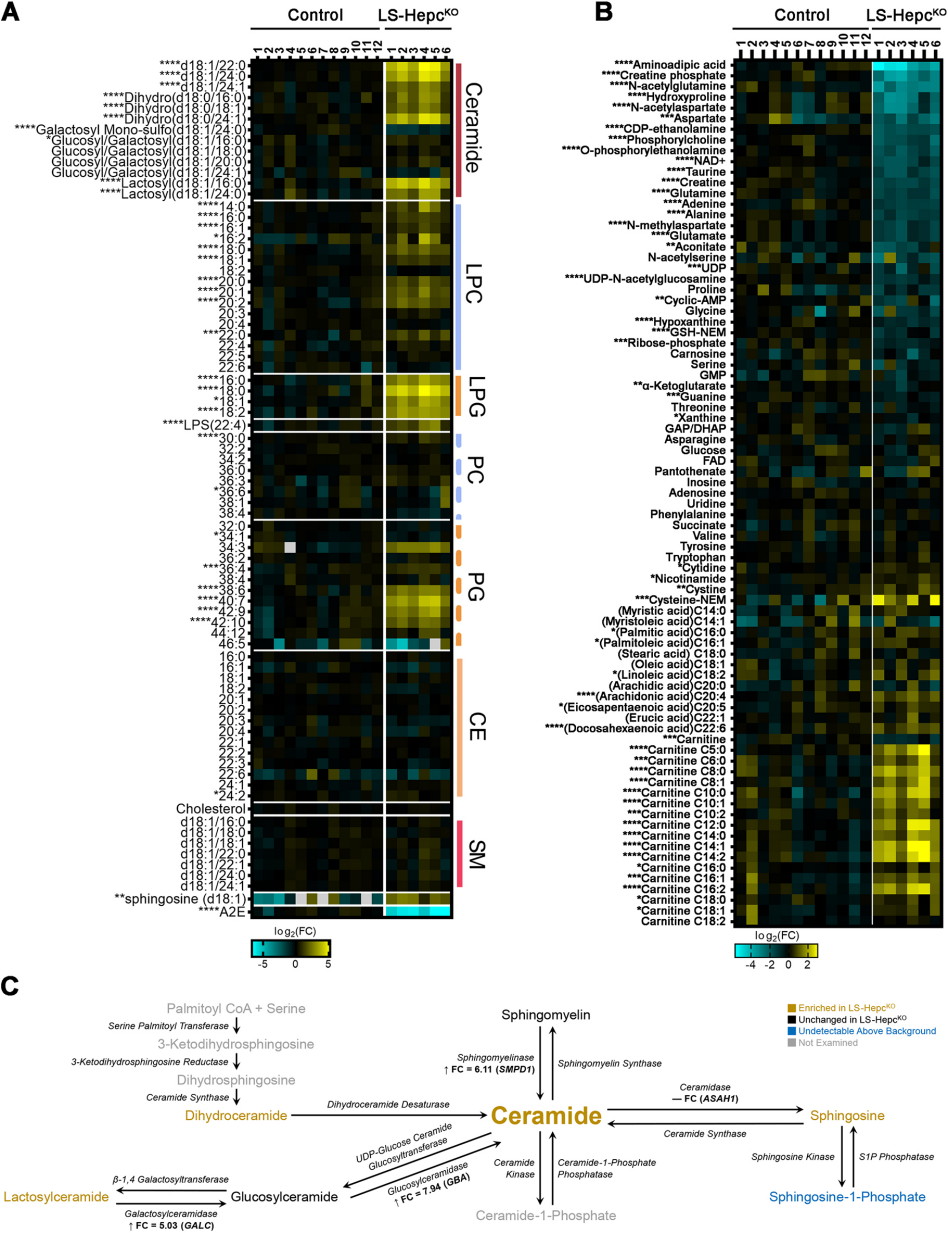
**LS-*Hepc*^KO^ RPE accumulates ceramides, acyl carnitines and DHA.** (A) Heatmap of a lipidomics experiment on isolated RPE from mice between 9 and 13 months old. Sphingosine was detected by HILIC-LCMS. (B) Metabolomics on isolated RPE from mice between 9 and 13 months old. Control mice were wild type. CE, cholesterol ester; LPC, lysophosphatidylcholine; LPG, lysophosphatidylglycerol; PC, phosphatidylcholine; PG, phosphatidylglycerol; SM, sphingomyelin. *P<0.05; **P<0.01; ***P<0.005; ****P<0.001 (two-tailed unpaired Student's *t*-test). (C) Schematic of ceramide metabolism, with colors corresponding to enrichment in LS-*Hepc*^KO^ versus control RPE. Lysosomal proteins detected by proteomics are indicated along with fold-change values, with positive values and up arrows indicating enrichment in LS-*Hepc*^KO^ RPE.

To further characterize the lipid composition of the lipofuscin of the LS-*Hepc*^KO^ RPE, we conducted liquid chromatography-mass spectrometry (LCMS)-based lipidomics and metabolomics on isolated RPE from LS-*Hepc*^KO^ mice aged 9-13 months (Fig. 6A,B; Tables S3 and S4). Several classes of lipids were significantly elevated in the LS-*Hepc*^KO^ RPE compared to age-matched wild-type RPE, including ceramides and lactosylceramides, LPCs, lysophosphatidylglycerols and phosphatidylglycerols. The bisretinoid A2E, a major component of mouse retinal lipofuscin and human peripheral retinal lipofuscin, was significantly depleted in the LS-*Hepc*^KO^ RPE, possibly due to conversion into oxidized products. We found accumulation of docosahexaenoic acid (DHA) (Fig. 6B), a major component of photoreceptor outer segment membranes, suggesting that outer segment phagocytosis contributes to lipid accumulation in the LS-*Hepc*^KO^ RPE and that the processing of this phagocytosed material is incomplete. The RPE of 12-month-old LS-*Hepc*^KO^ mice was further enriched in acyl carnitines while being depleted in carnitines.

Ceramides were significantly enriched in LS-*Hepc*^KO^ RPE, with a fold change ranging from 4 to 20 depending on the fatty acid. Dihydroceramides and lactosylceramides, which are other metabolites in ceramide metabolism, were also significantly enriched in the LS-*Hepc*^KO^ RPE, with a fold change from 4 to 14 and from 6 to 14, respectively. Sphingosine, detected by metabolomics (Fig. 6A), was significantly enriched in the LS-*Hepc*^KO^ RPE, although the fold change was lower at 4. In contrast to these enriched compounds, we did not detect significant differences in the levels of sphingomyelins and most glucosylceramides between control and LS-*Hepc*^KO^ RPE. Fig. 6C summarizes our lipidomics and proteomics results (Fig. 4) together in the context of ceramide metabolism and suggests that upregulation of sphingomyelinase and glucosylceramidase may contribute to the ceramide accumulation in the LS-*Hepc*^KO^ RPE.

### LS-*Hepc*^KO^ RPE lysosomes accumulate lipid peroxidation products

**Fig. 7.**
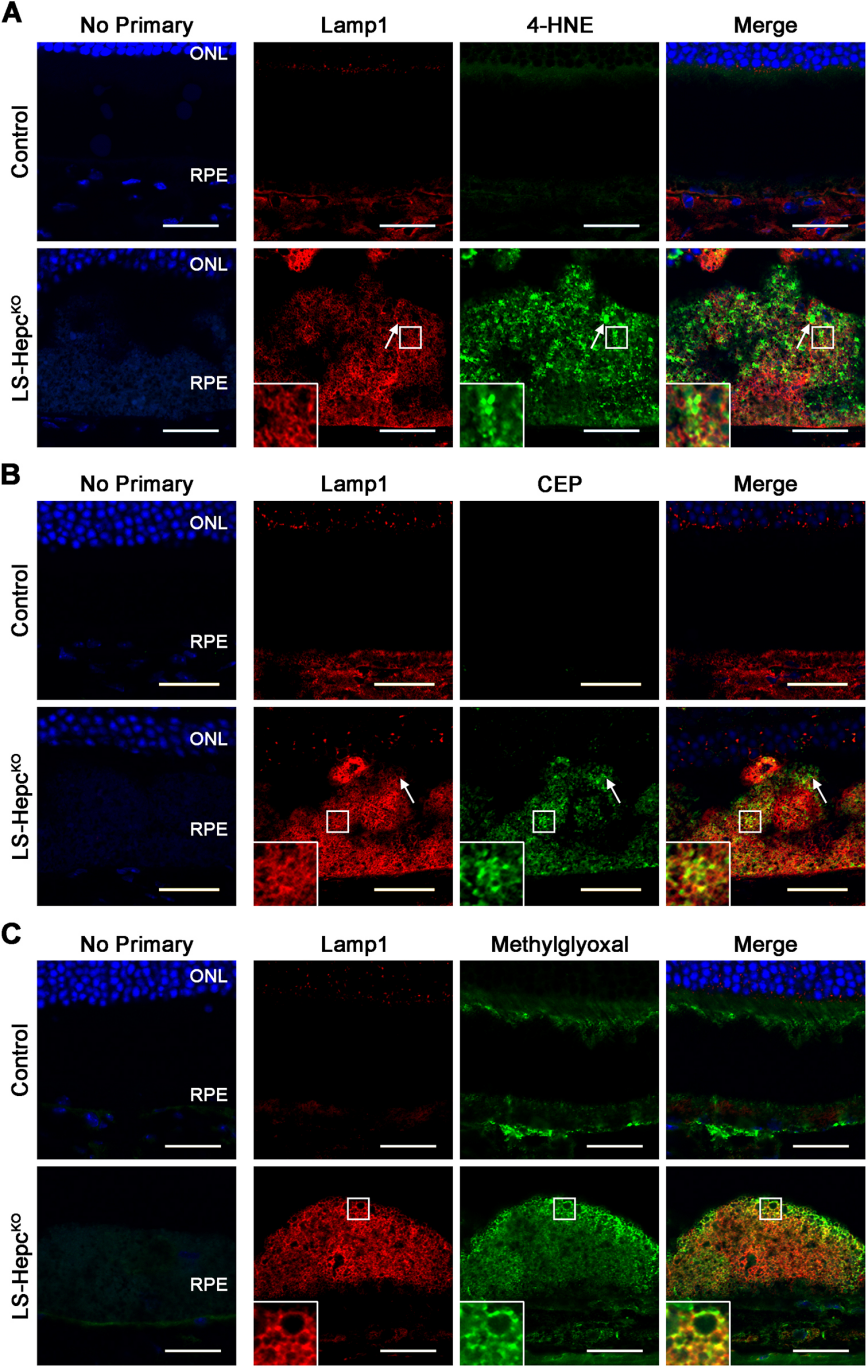
**LS-*Hepc*^KO^ RPE lysosomes accumulate lipid peroxidation products.** (A-C) Representative confocal images of immunofluorescence for Lamp1 (red) and 4-hydroxynonenal (4-HNE; green) (A), carboxyethylpyrrole (CEP; green) (B) or methylglyoxal (green) (C) in 12-month-old mice. Regions bounded by white squares are enlarged in the insets. White arrows indicate an example of colocalization between Lamp1 and 4-HNE (A) or CEP (B). Nuclei are labeled with DAPI (blue). All control mice were wild type. Representative immunolabeling images are shown from *n*=4 mice per genotype. ONL, outer nuclear layer; RPE, retinal pigment epithelium. Scale bars: 25 μm.

Iron is known to catalyze the formation of lipid peroxidation products via the Fenton reaction (Gutteridge, 1986; Minotti and Aust, 1987). To determine whether iron led to lipid peroxidation in the LS-*Hepc*^KO^ RPE, we used IHC to examine levels of 4-HNE and CEP. 4-HNE immunoreactivity was greatly increased in the LS-*Hepc*^KO^ RPE compared to the control RPE (Fig. 7A). Some of the 4-HNE-positive regions were outlined by Lamp1-positive membranes (white arrows), consistent with intralysosomal accumulation of 4-HNE. 4-HNE signals were also localized to the lysosomal membrane or surrounding areas. CEP immunoreactivity was similarly increased in the LS-*Hepc*^KO^ RPE relative to the control RPE (Fig. 7B) and featured a localization pattern similar to that of 4-HNE.

Given the role of oxidative stress in producing advanced glycation end products, we examined the levels of the dicarbonyl methylglyoxal, a highly potent glycating agent (Rabbani and Thornalley, 2008), in the LS-*Hepc*^KO^ RPE. Methylglyoxal naturally forms from glycolysis intermediates (Allaman et al., 2015) and oxidation of ascorbic acid (Fan et al., 2020; Nemet and Monnier, 2011), and, notably, can also be generated from iron-induced oxidation or photodegradation of the bisretinoid A2E (Ueda et al., 2018; Yoon et al., 2012). We found significant immunolabeling of methylglyoxal in the LS-*Hepc*^KO^ RPE (Fig. 7C). Methylglyoxal immunolabeling colocalized strongly with that of Lamp1, consistent with lysosomal membrane accumulation. There was much greater immunolabeling at the apical aspect of RPE cells, suggesting methylglyoxal formation from photoreceptor-derived bisretinoids. This is consistent with the depletion of A2E in the LS-*Hepc*^KO^ RPE (Fig. 6A). These data suggest that the accumulation of lipid peroxidation products may underlie the lysosomal pathology and lipid accumulation that are seen in the LS-*Hepc*^KO^ model.

### Aged LS-*Hepc*^KO^ RPE undergoes cell death and displays AMD morphologies

**Fig. 8.**
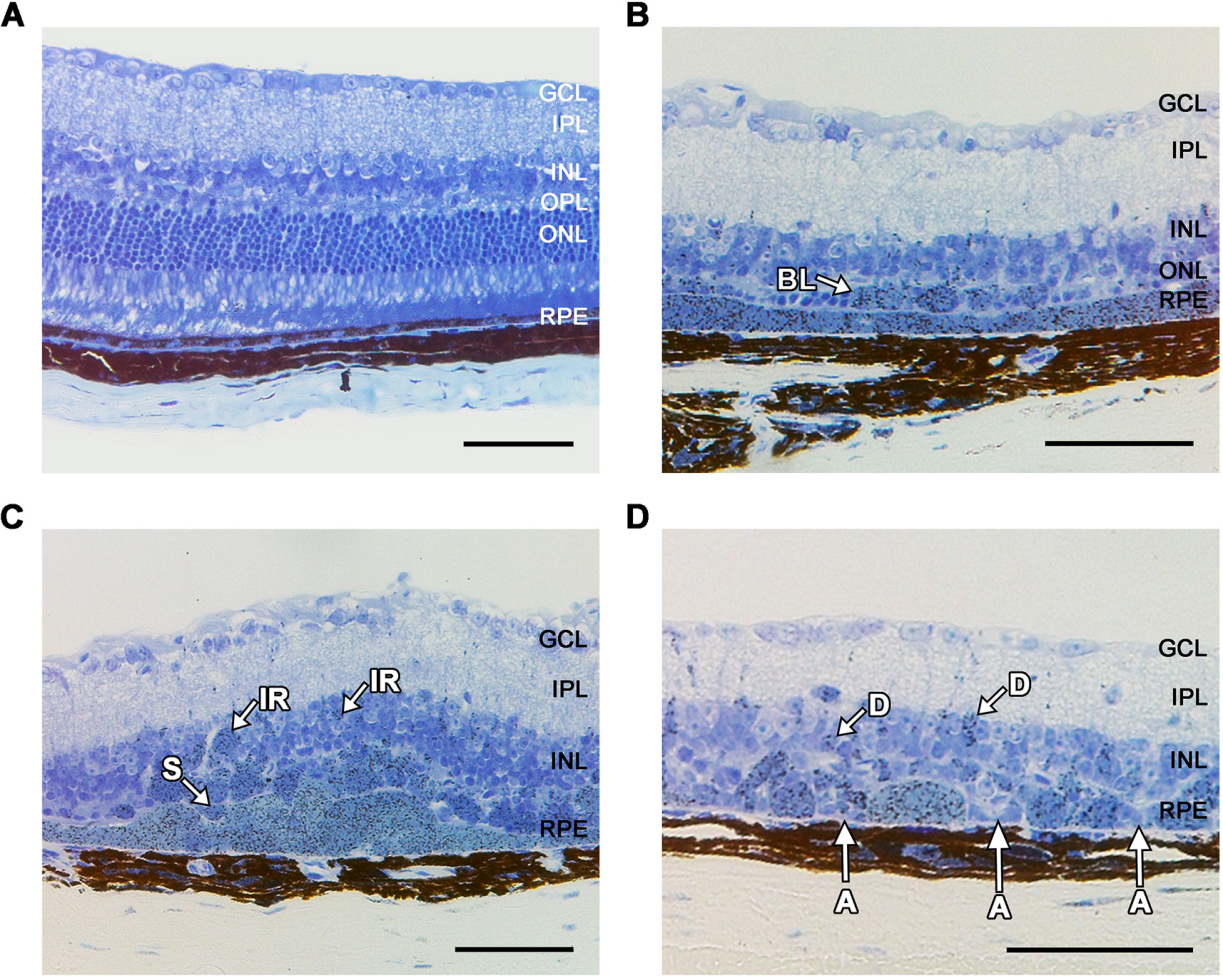
**Aged LS-*Hepc*^KO^ RPE undergoes cell death and displays AMD morphologies.** (A-D) Representative images of Toluidine Blue-stained plastic sections in 16-month-old control *AlbCre^−^*, *Hepc*^f/f^ (A) and LS-*Hepc*^KO^ (B-D) mice demonstrating regions of hypertrophic RPE and several of the RPE phenotypes seen in AMD. Non-uniform and very non-uniform phenotypes are seen in all images. Morphologies shown are: A, atrophy without basal laminar deposits; D, dissociated; BL, bilaminar; IR, intraretinal; S, sloughed; Control mice were wild type. GCL, ganglion cell layer; INL, inner nuclear layer; IPL, inner plexiform layer; ONL, outer nuclear layer; OPL, outer plexiform layer; RPE, retinal pigment epithelium. Scale bars: 25 μm.

Lipid peroxidation products are cytotoxic, possibly by inducing LMP (Boya and Kroemer, 2008). To investigate whether LMP in the LS-*Hepc*^KO^ RPE leads to cell death, we aged LS-*Hepc*^KO^ mice to 16 months and examined retinal morphology (Fig. 8). Patches of hypertrophic RPE were observed throughout the retina, similar to the retinas of 12-month-old LS-*Hepc*^KO^ mice, although there were also areas of atrophic and missing RPE that presumably reflect cells that had died. The neural retina was thinner over these areas of missing RPE. Notably, the RPE in LS-*Hepc*^KO^ mice also displayed some of the pathologic RPE morphologies seen in AMD, including ‘non uniform,’ ‘very non-uniform,’ ‘sloughed,’ ‘bilaminar,’ ‘intraretinal’ and ‘dissociated’ RPE (Zanzottera et al., 2015). These findings suggest a gradual progression wherein RPE iron overload catalyzes lipid peroxidation product formation, which may contribute to lysosomal dysfunction and LMP that ultimately causes RPE cell death and development of AMD-like pathology.

## DISCUSSION

There is currently no treatment for advanced dry AMD, also called geographic atrophy. Dietary changes and vitamin/antioxidant supplementation are moderately helpful in slowing the progression of early AMD (Age-Related Eye Disease Study Research Group, 2001). These interventions can directly modulate retinal iron levels or mitigate the effects of iron overload. We have previously demonstrated that iron accumulates in the RPE of patients with AMD (Hahn et al., 2003). Animal models of RPE iron overload also display wide-spaced collagen deposits similar to those in AMD retinas (Hahn et al., 2004). Complement C3, which has been found in drusen (Anderson et al., 2002), is upregulated by iron loading in cultured RPE cells and in mice with RPE iron overload (Li et al., 2015). Despite this evidence associating iron accumulation and oxidative stress to AMD pathogenesis, the mechanisms behind these findings were unknown.

In this study, we demonstrate that iron can induce a cell-autonomous lysosomal phenotype in iPS-RPE cells by causing lysosomal accumulation and disrupting lysosomal enzyme function (Fig. 1). We also find evidence of *in vivo* lysosomal dysfunction in the LS-*Hepc*^KO^ mouse RPE. We identify the inclusions filling the LS-*Hepc*^KO^ RPE as lysosomes (Figs 3 and 4) and show that they exhibit LMP (Fig. 3C), have impaired cathepsin D maturation (Fig. 3D) and accumulate various neutral lipids and lipid peroxidation products (Figs 5–7). The lipid peroxidation products and LMP ultimately lead to cell death (Fig. 8). Impaired lysosomal function also reduces autophagic clearance, as demonstrated through the enrichment of autophagosome markers (Fig. 3D; Table S2), leading to lysosomal accumulation. These studies point to a mechanism by which RPE iron overload induces the accumulation of lipid-laden, dysfunctional lysosomes that are likely to leak cytotoxic enzymes, lead to RPE phenotypes seen in AMD (Fig. 8) and eventually contribute to cell death.

We incubated iPS-RPE cells with iron for 6 and 9 weeks to model the gradual iron accumulation in AMD. Iron overload induced an accumulation of pro-cathepsin D and the 31 kDa form of cathepsin B (Fig. 1A). In contrast to the findings of other studies that demonstrated an iron-induced decrease in the activity of cathepsins D and B (Chen et al., 2009a; Voisin et al., 2019), we found that iron treatment did not change cathepsin D activity and surprisingly increased cathepsin B activity (Fig. 1F,G). The difference in our results could be explained by our use of FeSO_4_ rather than other iron-containing compounds, our use of a lower concentration of iron and, most likely, our long incubation time of 6 weeks. Our chronic and lower dose of iron provided a mild insult that may not have completely inactivated lysosomal enzymes, while also providing time for lysosomal biogenesis. This is consistent with the increased LysoTracker staining in iron-loaded cells, reflecting increased lysosomal number (Fig. 1B,C), which also occurs in macrophages exposed to iron (Kao et al., 2016). An increased number of lysosomes could also explain the decreased pH in iron-loaded cells (Fig. 1E).

Despite the lack of change in cathepsin D activity and increase in cathepsin B activity, iron induced a generalized deficit in proteolysis based on a decrease in the number of DQ-BSA puncta per cell (Fig. 1B,D). This suggests that lysosomal proteases other than cathepsin D and B contribute to DQ-BSA cleavage and are impaired by iron. Iron may impact lysosomal enzymes heterogeneously based on the preferential binding of lipid peroxidation products to histidine and cysteine residues (Esterbauer et al., 1991; Uchida, 2003). Indeed, out of the three other enzymes for which we measured the activity in iron-loaded iPS-RPE, lysosomal acid lipase and acid sphingomyelinase exhibited decreased activities, whereas glucosylceramidase activity was not impacted (Fig. 1H-J). Cysteine and histidine residues are both critical for the enzymatic function of lysosomal acid lipase (Lohse et al., 1997a,b), and the active site of acid sphingomyelinase is rich in histidine (Zhou et al., 2016). In contrast, neither amino acid appears critical for the function of glucosylceramidase, which relies instead on key glutamic acid residues (Fabrega et al., 2000). A caveat of the DQ-BSA assay is that there is no way to rule out impaired uptake of the DQ-BSA into iron-overloaded cells or their lysosomes.

Iron overload leads to the production of 4-HNE, CEP and methylglyoxal *in vivo* (Fig. 7). These lipid peroxidation products likely bind to the active site of lysosomal cathepsins in the LS-*Hepc*^KO^ RPE (Krohne et al., 2010), inhibiting their ability to cleave pro-cathepsin D into the mature form (Hoppe et al., 2004) and thereby increasing the ratio of pro-cathepsin D to mature cathepsin D (Fig. 3D; Fig. S3). Iron-catalyzed lipid peroxidation products may similarly be responsible in inducing LMP (Cheng et al., 2019; Rajapakse et al., 2017). Whereas immunolabeling of Lamp1 produced well-resolved structures in the LS-*Hepc*^KO^ RPE, cathepsin D (Fig. 3B), 4-HNE (Fig. 7A) and CEP (Fig. 7B) appeared to be cytoplasmic, suggesting leakage of lysosomal contents. We found strong galectin-3 colocalization with Lamp1 in a large subset of LS-*Hepc*^KO^ RPE cells (Fig. 3C), constituting a sensitive measure of LMP (Aits et al., 2015). In contrast, autofluorescence and BODIPY 493/503 staining appeared to be present entirely within the lysosome (Fig. 5), suggesting that lipids may be unable to translocate out of the lysosome owing to their size or hydrophobicity.

Uncontrolled LMP is ultimately cytotoxic and has recently been shown to be triggered by lipofuscin and induce necroptosis in RPE cells (Pan et al., 2021). LMP involves the release of cytosolic cathepsins, which activate the pro-apoptotic protein Bid, cleave the main caspase inhibitor XIAP, and cleave multiple members of the anti-apoptotic Bcl-2 family of proteins (e.g. Bcl-2, Bcl-X_L_ and Mcl-1) (Boya and Kroemer, 2008; Droga-Mazovec et al., 2008). This in turn leads to mitochondrial outer membrane permeabilization, cytochrome c release and caspase activation. Our proteomics analysis further suggests activation of this cell death pathway, as both the pro-apoptotic proteins Bid (log_2_FC=1.31) and Bax (log_2_FC=0.76) were enriched in the LS-*Hepc*^KO^ RPE (Table S2). Significantly, the caspase inhibitor Xiap was the second-most depleted protein in our study (log_2_FC=−3.06). The time course of LMP-mediated cell death is variable, with numerous cellular compensatory mechanisms. Cystatins (cysteine protease inhibitors) and serpins (serine protease inhibitors) can both inactivate cytosolic cathepsins (Boya and Kroemer, 2008). We found evidence of cellular compensation, with enrichment of cystatin B (Cstb) (log_2_FC=0.98) and numerous serpins in the LS-*Hepc*^KO^ RPE (Table S2), although the role of these particular serpins in LMP is unknown. These compensatory mechanisms may explain why evidence of cell death was observed only after 16 months (Fig. 8). This mechanism of cell death is notably distinct from ferroptosis. Ferroptosis involves iron-induced lipid peroxidation similar to what we observed (Stockwell, 2022), but it also involves alterations in iron influx and efflux that are designed to kill the cell. Gpx4 is downregulated in ferroptosis (Seibt et al., 2019), but it is highly enriched in the LS-*Hepc*^KO^ RPE (log_2_FC=2.20), suggestive of attempted cellular compensation against iron-mediated lipid peroxidation.

Damaged lysosomes can be cleared by lysophagy (Eapen et al., 2021), but as functional lysosomal enzymes are required to clear autophagic cargo, lysosomal dysfunction can also impair lysophagy. We found evidence of impaired autophagosome clearance, as evidenced by the accumulation of p62 and LC3B-II (Fig. 3D). Similarly, our proteomics analysis revealed enrichment of p62 (log_2_FC=2.41) and LC3B (log_2_FC=0.94) in the LS-*Hepc*^KO^ RPE (Table S2). Cells may compensate for lysosomal damage through upregulation of lysosomal biogenesis via the mTORC1-TFEB pathway (Wang et al., 2018). TFEB is the master regulator of the coordinated lysosomal expression and regulation (CLEAR) gene network, with TFEB nuclear translocation associated with increased lysosomal biogenesis (Palmieri et al., 2011). We did not detect significant upregulation of CLEAR network genes in an RNA sequencing study we performed comparing the LS-*Hepc*^KO^ to control RPE [data available at Gene Expression Omnibus (GEO) (accession GSE237309)], suggesting that lysosomal biogenesis is maintained but not augmented in 12-month-old LS-*Hepc*^KO^ RPE to provide a basal level of functional lysosomes. The accumulation of autophagosomes without increased lysosomal biogenesis suggests that the lysosomal accumulation is owing to decreased lysophagy.

The ceramide accumulation in the LS-*Hepc*^KO^ RPE is of particular interest given the role of ceramide in signaling, lysosomal localization of many enzymes in ceramide metabolism and accumulation of ceramide in retinal disease (Simón et al., 2019). High levels of ceramide may have a causal role in LMP, as chemical inhibition of acid ceramidase (ASAH1) correlates with LMP *in vitro* in both lymphocytes and A549 cells (Taniguchi et al., 2015; Yamane et al., 2017). In concordance with ceramide accumulation seen in the LS-*Hepc*^KO^ RPE, we found significantly increased protein levels of glucosylceramidase (log_2_FC=2.99) and acid sphingomyelinase (log_2_FC=2.61), both of which would increase ceramide generation (Figs 4F and 6C). However, we observed no change in glucosylceramidase activity and decreased acid sphingomyelinase activity with iron in our iPS-RPE model (Fig. 1H,I). This may reflect the differences between the *in vivo* and *in vitro* models. It is possible that acid ceramidase (ASAH1) also plays a role in ceramide accumulation in LS-*Hepc*^KO^ RPE. Decreasing ASAH1 activity led to oxidative stress-induced ceramide accumulation in the placenta (Melland-Smith et al., 2015), and ASAH1 overexpression in ARPE-19 cells mitigated ceramide accumulation caused by hydrogen peroxide-induced oxidative stress (Sugano et al., 2019). Although we were unable to examine acid ceramidase activity owing to a lack of currently available methods, ASAH1 protein levels were not substantially different between LS-*Hepc*^KO^ and control RPE. Furthermore, sphingosine levels were only mildly elevated in the LS-*Hepc*^KO^ RPE despite dramatic ceramide accumulation (Fig. 6A), which would be consistent with decreased acid ceramidase activity.

The accumulation of LPCs in LS-*Hepc*^KO^ RPE is also of note, given their well-known association with oxidized low-density lipoprotein (LDL) (Law et al., 2019). Compared to normal LDL, oxidized LDL contains a fivefold increase in LPC and accounts for up to half of the phosphatidylcholine equivalents in oxidized LDL (Choi et al., 2011; Schmitz and Ruebsaamen, 2010). LPC can be produced enzymatically from oxidized LDL by lipoprotein-associated phospholipase A2 (Lp-PLA_2_) (Schmitz and Ruebsaamen, 2010) and is degraded into lysophosphatidic acid by autotaxin (Nakanaga et al., 2010). Lp-PLA_2_ (encoded by *Pla2g7*) is the 27th-most enriched protein in the LS-*Hepc*^KO^ RPE (log_2_FC=3.44), whereas autotaxin (encoded by *Enpp2*) is the third-most depleted protein (log_2_FC=−3.03), consistent with the increase in LPC. LPC can additionally be generated non-enzymatically through the peroxidation of DHA into 4-hydroxy-7-oxo-5-heptenoic acid (HOHA) (Choi et al., 2011). Given that HOHA reacts with proteins to form CEP adducts, which we have demonstrated to accumulate in the LS-*Hepc*^KO^ RPE (Fig. 7B), it is likely that both the enzymatic and non-enzymatic mechanisms contribute to the LPC content of the LS-*Hepc*^KO^ RPE.

Although we have comprehensively examined the lipid profile of the LS-*Hepc*^KO^ RPE in this study, the identity of the autofluorescent materials in the lysosome is still unknown. The bisretinoid A2E is an unlikely candidate, as we show here that the LS-*Hepc*^KO^ RPE has diminished levels of A2E, corroborating a previous finding that measured A2E in these mice by a different method (Zhao et al., 2021). The A2E depletion could be consistent with recent findings calling into question the historic association of A2E with AMD, such as the discovery of higher levels of A2E in the peripheral retina rather than in the macula (Kotnala et al., 2022), and may reflect A2E conversion into oxidized byproducts by iron. The accumulation of methylglyoxal in the LS-*Hepc*^KO^ RPE (Fig. 7C) suggests that bisretinoid oxidation indeed occurs in this model. In addition to oxidized bisretinoids, DHA oxidation products are likely also major contributors to the autofluorescence, as DHA is highly abundant in photoreceptor outer segments and would presumably be intralysosomal at some point during its metabolic cycling. Although DHA recycling to the photoreceptors generally occurs efficiently, the mechanisms of DHA recycling are incompletely understood, and dysfunctional lysosomes may prevent this recycling. Oxidation of DHA would lead to the production of CEP adducts, which we have shown to accumulate in the LS-*Hepc*^KO^ RPE. Consistent with the deleterious effects of these adducts, our laboratory has demonstrated that feeding mice a diet containing deuterated DHA, which inhibits DHA peroxidation due to the kinetic isotope effect (Firsov et al., 2019; Shchepinov, 2020), can completely protect the RPE and photoreceptors from damage caused by intravitreal iron injections (Liu et al., 2022).

Our data suggest a mechanism by which iron induces the accumulation of lipid-laden lysosomes in LS-*Hepc*^KO^ mice. Through the Fenton reaction, iron catalyzes the formation of lipid peroxidation products, which bind to and inactivate lysosomal enzymes. This leads to the accumulation of neutral lipids, including ceramides. The lipid peroxidation products and ceramides can both induce LMP, cause RPE de-differentiation and eventually lead to RPE cell death. Compensatory mechanisms such as lysophagy are initiated to remove the damaged lysosomes but, given broad enzyme inactivation, cannot be completed. This failure in lysophagy, together with unchanged lysosomal biogenesis, leads to the massive lysosomal accumulation seen in the LS-*Hepc*^KO^ RPE before the cells ultimately die.

The RPE in the LS-*Hepc*^KO^ mouse develops many of the RPE morphologies observed in AMD (Fig. 8) and also has additional features of AMD, including RPE hypertrophy and decreased photoreceptor function (Baumann et al., 2019). The LS-*Hepc*^KO^ RPE furthermore accumulates galectin-3, which is the most increased protein in Bruch's membrane of patients with advanced AMD (Yuan et al., 2010), as well as neutral lipids, which accumulate in the RPE and Bruch's membrane with aging (Curcio et al., 2009). These data suggest that oxidative stress may contribute to the pathogenesis of AMD by causing lysosomal dysfunction and the accumulation of lipid-laden lysosomes in the human RPE. This mechanism may also be generalized to other diseases, as our proteomics analysis found association of the enriched genes in the LS-*Hepc*^KO^ RPE with atherosclerosis, cardiovascular disease and diabetes, in addition to AMD (Fig. S4). The role of iron-induced oxidative stress in these other diseases is well established (Nankivell et al., 1994; Satchell and Leake, 2012). These examples suggest that reducing iron levels, while avoiding iron deficiency, by dietary changes, chelation (Song et al., 2014), reduction in inflammation (Sterling et al., 2022) or administration of oxidation-resistant lipids (Liu et al., 2022) might prevent the accumulation of lipid-laden lysosomes and improve the treatment for AMD and numerous other common diseases.

## MATERIALS AND METHODS

### Generation of iPS-RPE cells

iPS cells (iPSCs) (Penn iPSC Core, University of Pennsylvania) were thawed and cultured in StemMACS iPS-Brew XF medium (Miltenyi Biotec) on Matrigel-coated plates at 37°C, 5% O_2_ and 5% CO_2_. After checking the pluripotency markers for stemness (SSEA-4 and TRA-1-60) using flow cytometry, RPE differentiation was initiated with iPSCs having reached 50-60% confluency according to a previously established protocol (Duong et al., 2018). Briefly, the plates were transferred to a normal incubator (37°C and 5% CO_2_) for RPE induction with base medium [Dulbecco's modified Eagle medium (DMEM)/F12 (Thermo Fisher Scientific) with 2% B27, 1% N2, 1× penicillin-streptomycin, and 1× GlutaMAX] for 0-14 days. For the first 2 days (days 0 and 1), the cells were treated daily with Noggin (50 ng/ml; R&D Systems), DKK1 (10 ng/ml; R&D Systems), IGF1 (10 ng/ml; R&D Systems) and nicotinamide (10 mM; Sigma-Aldrich). The cells were treated with Noggin (50 ng/ml), DKK1 (10 ng/ml), IGF1 (10 ng/ml) bFGF2 (5 ng/ml; R&D Systems) and nicotinamide (10 mM) daily on days 2 and 3. For the next 2 days (days 4 and 5), cells were treated with activin A (100 ng/ml; R&D Systems), IGF1 (10 ng/ml) and DKK1 (10 ng/ml). From days 6 to 14, the medium was supplemented daily with activin A (100 ng/ml), SU5402 (5 μM; Selleck Chemicals) and vasoactive intestinal peptide (VIP) (1 nM; Sigma-Aldrich). From day 15 onwards, the cells were maintained in DMEM/Ham's F12 (70:30) (Thermo Fisher Scientific), 2% B27, 1× GlutaMAX and 1× penicillin-streptomycin (RPE medium) for the next 21 days until the appearance of cobblestone-shaped RPE cells. At day 35, the cells were dissociated using Accutase (Sigma-Aldrich)/DNase1 (EMD Millipore) for 30 min and passaged into Matrigel-coated plates in RPE medium containing the ROCK inhibitor thiazovivin (5 μM; Tocris Bioscience). After cell attachment, the RPE medium was replaced with medium without thiazovivin and changed every 2-3 days.

### Culture of ARPE-19 cells

ARPE-19 cells were obtained from American Type Culture Collection and cultured in DMEM/F12 with 10% fetal bovine serum (Cytiva) as previously described (Dunn et al., 1996).

### *In vitro* iron loading

Cells were maintained in RPE medium containing 50 or 100 μM FeSO_4_. These concentrations are higher than normal serum values of 8-24 μM (Hetet et al., 2003) but much lower than the 500-2000 μM concentrations used for other studies of iron in the RPE (Chen et al., 2009a), and are therefore more likely to resemble *in vivo* iron overload. The medium also contained 1 mM sodium ascorbate, resembling the ascorbate concentration in the human eye (Shui et al., 2009; Takano et al., 1997), to keep the iron in the ferrous state. The medium was refreshed every other day.

### *In vitro* lysosome characterization

iPS-RPE cells were incubated with 10 μg/ml DQ Green Bovine Serum Albumin (BSA) (Thermo Fisher Scientific) diluted in RPE medium for 3 h at 37°C. DQ Green BSA is a substrate that emits fluorescence upon cleavage by cellular proteases. Cells were subsequently washed with PBS and incubated with 50 nM LysoTracker Red DND-99 (Thermo Fisher Scientific) diluted in RPE medium for 30 min at 37°C. Cells preincubated with 200 nM bafilomycin (Sigma-Aldrich) to inhibit lysosomal acidification served as a control. Following incubation with LysoTracker, cells were washed with PBS, fixed with 4% paraformaldehyde (PFA) for 15 min, and mounted in Fluoromount-G with DAPI (SouthernBiotech) for nuclear staining. Images were captured with a Nikon A1R laser scanning confocal microscope. LysoTracker- and DQ Green BSA-positive puncta were counted using ImageJ (Schneider et al., 2012), and cell borders were determined using LysoTracker background fluorescence. A minimum of three images per treatment group were analyzed, with an average of 300 cells counted per image.

### Lysosomal pH measurements

Lysosomal pH measurements were obtained in iPS-RPE cells using LysoSensor Yellow/Blue DND-160 (Thermo Fisher Scientific) as previously described (Baltazar et al., 2012). Cells were treated with 10 μM tamoxifen to serve as a positive control for lysosomal alkalinization.

### Lysosomal enzyme activity assays

Lysosomal enzyme activity in iPS-RPE cells cultured in 12-well plates was assessed with the kits for the following enzymes: cathepsin D (Abcam, ab65302), cathepsin B (Abcam, ab65300), acidic sphingomyelinase (Abcam, ab190554), glucosylceramidase (Abcam, ab273339) and LysoLive lysosomal acid lipase (Abcam, ab253380). Assays were performed following the manufacturer's protocols. For the cathepsin D, cathepsin B, acidic sphingomyelinase and glucosylceramidase assays, whole-cell protein lysates were used with results normalized to the protein concentration determined by Bradford assays (Bio-Rad).

### Animals

AlbCre^+^, *Hepc*^f/f^ (LS-*Hepc*^KO^) and AlbCre^−^, *Hepc*^f/f^ (control) mice were generated as previously described (Baumann et al., 2019). Wild-type C57BL/6J mice were also used owing to limited numbers of the AlbCre^−^, *Hepc*^f/f^ genotype; there is no structural difference between AlbCre^−^, *Hepc*^f/f^ and wild-type mice based on *in vivo* imaging. All mice were housed in standard conditions at 26°C with water and fed *ad libitum* with a diet containing approximately 300 ppm of iron, the amount of iron most commonly used in rodent chows. Mice were maintained in cyclic light conditions with a 12 h:12 h light-dark cycle. All mice were on a C57BL/6J background, confirmed negative for *rd1* and *rd8*. Only males were used in this study, as females of the AlbCre^+^, *Hepc*^f/f^ genotype develop retinal degeneration much more slowly (Baumann et al., 2019). Experimental procedures were performed in accordance with the Association for Research in Vision and Ophthalmology (ARVO) statement for the use of animals in ophthalmology and vision research. All protocols were approved by the University of Pennsylvania Animal Care and Use Committee.

### *In vivo* imaging system

*In vivo* imaging of mice was performed as previously described (Liu et al., 2021). Briefly, optical coherence tomography imaging was performed using a Bioptigen Envisu (R2200, Bioptigen) coupled to a broadband LED light source (T870-HP, Superlum Diodes), which enables ultra-high-resolution imaging. Confocal scanning laser ophthalmoscopy was performed using the Spectralis HRA imaging platform (Heidelberg Engineering).

### Dissection of murine RPE

Mice were euthanized by ketamine/xylazine injection followed by cervical dislocation. Eyes were immediately enucleated. Following enzymatic digestion with dispase (Thermo Fisher Scientific) and hyaluronidase (Sigma-Aldrich), the neural retina was dissected away and the RPE was subsequently isolated mechanically to obtain a population of cells that is highly enriched for the RPE, as previously described (Hadziahmetovic et al., 2012).

### Quantitative real-time PCR

RNA was isolated with an RNeasy kit (QIAGEN) following the manufacturer's protocol and cDNA was synthesized using reverse transcription reagents (TaqMan). Quantitative real-time PCR (qPCR) was used to analyze expression of *BEST1*, *MITF*, *RLBP1* and *RPE65*, as previously described (Hadziahmetovic et al., 2011). TaqMan gene expression assays were used for PCR, with *GAPDH* serving as the internal control. qPCR was performed using an ABI Prism 7500 (Applied Biosystems), with all reactions performed in technical triplicates. The following probes were used: *BEST1* (Hs00188249_m1), *MITF* (Hs01117294_m1), *RLBP1* (Hs00165632_m1) and *RPE65* (Hs01071462_m1).

### Western blot analysis

Lysates were generated from isolated murine RPE and iPS-RPE cells by lysis with RIPA buffer (Cell Signaling Technology) supplemented with cOmplete protease inhibitor cocktail (Roche). Samples were treated and run as previously described (Li et al., 2015). Protein concentration was determined using the Bradford assay (Bio-Rad). The primary and secondary antibodies used are listed in Table S1. AzureRed fluorescent total protein stain (Azure Biosystems) was used for protein normalization. Imaging was done using the GE Amersham Imager 600. Band densitometry was performed without prior image manipulation using ImageJ (Schneider et al., 2012).

### Morphological analysis

Morphological analysis of murine retinas was performed as previously described (Hadziahmetovic et al., 2011). Briefly, mice were aged to 16 months and enucleated eyes were fixed overnight in 2% PFA/2% glutaraldehyde. Eyes were embedded in glycol methacrylate (Polysciences), cut into 3 μm sections using a microtome (Leica CM3050S), and stained with Toluidine Blue. Sections were imaged using the Nikon Elements software. Images were captured using a Nikon 80i microscope with a 40× objective, total 400× magnification and 0.75 aperture. The DS-Fi2 camera was used with the Nikon Elements software. Image intensity levels were adjusted uniformly across all photos within each experiment using Adobe Photoshop.

### Fixation of eyes and immunofluorescence

Mice were aged to 9-12 months and euthanized as described above. Fixation of eyes and preparation of eyecups were performed as previously described (Shu et al., 2019). Briefly, the cornea and lens were dissected away, and the resulting eyecups were fixed in 4% PFA for 15 min, dehydrated in 30% w/v sucrose overnight, and subsequently embedded in a mixture of polyvinyl alcohol and polyethylene glycol (Tissue-Tek O.C.T. compound, Sakura). Immunofluorescence was performed on 10 μm-thick cryosections as previously described (Hadziahmetovic et al., 2011). Sections were permeabilized and blocked for 1 h at room temperature using PBS containing 1% normal donkey serum, 1% BSA and 0.1% saponin or 0.1% Triton X-100. Sections were incubated overnight at 4°C with the primary antibody diluted in PBS containing 1% BSA and 0.1% saponin or 0.1% Triton X-100. Primary and secondary antibodies used are listed in Table S1. Control sections from wild-type mice were treated identically with the omission of the primary antibody. All sections were cut in the sagittal plane, and sections chosen for labeling were close to the optic disc. Following primary antibody incubation, sections were rinsed with PBS three times and subsequently incubated with the secondary antibody for 1 h at room temperature. Nuclei were stained with Hoechst 33258 (Sigma-Aldrich). Sections were imaged by fluorescence microscopy with identical exposure parameters across genotypes using the Nikon Elements software. Images were captured using a Nikon 80i microscope or Nikon A1R laser scanning confocal microscope as previously described (Dhingra et al., 2018).

### Transmission EM

Mice were transcardially perfused with 10 ml of heparin saline (20 U/ml), 70 ml of 1% glutaraldehyde and 4% PFA in 0.1 M cacodylic acid buffer (pH 7.4). Eyecups were prepared and stored in 2.5% glutaraldehyde, 4% PFA in 0.1 M cacodylate buffer at 4°C as previously described (Chuang et al., 2022). Vibratome sections (120 µm thick) obtained from small pieces of eyecup (∼2×2 mm) embedded in 5% agarose II/0.1 M cacodylic acid buffer were processed for OTO *en bloc* staining. Specifically, after several washes in ice-cold 0.15 M cacodylate buffer containing 2 mM calcium chloride, specimens were incubated with 1.5% potassium ferrocyanide, 2 mM calcium chloride and 2% osmium tetroxide in 0.15 M cacodylate buffer, pH 7.4 for 1 h on ice. The sections were then treated with 10 mg/ml thiocarbohydrazide (Polysciences) solution for 20 min at room temperature, followed by 2% osmium tetroxide fixation for 30 min at room temperature. The *en bloc*-stained tissues were dehydrated with progressively higher ethanol concentrations and embedded in Epon. Ultrathin sections (72 nm) were collected on G400-Cu grids (Electron Microscopy Sciences) and were examined under a TECNAI microscope for transmission EM analysis.

### Neutral lipid staining

For neutral lipid staining, retinal cryosections were rehydrated in PBS and incubated with 10 μg/ml BODIPY 493/503 (Thermo Fisher Scientific) for 1 h at room temperature. BODIPY 493/503 staining was done following any relevant antibody incubation for immunofluorescence. Nuclei were labeled with Hoechst 33258. Sections were imaged using a Nikon A1R laser scanning confocal microscope.

### Proteomics

Isolated RPE cell pellets collected from five 12-month-old LS-*Hepc*^KO^ and six age-matched control (AlbCre^−^, *Hepc*^f/f^) mice were lysed, solubilized and digested with the iST kit (PreOmics, Martinsried, Germany) according to the manufacturer's protocol. This kit enables quick and robust sample preparation for mass spectrometry (MS) (Sielaff et al., 2017). Briefly, the resulting pellet was solubilized, reduced and alkylated by the addition of sodium deoxycholate buffer containing tris(2-carboxyethyl)phosphine (TCEP) and 2-chloroacetamide, then heated to 95°C for 10 min. Proteins were enzymatically hydrolyzed for 1.5 h at 37°C by addition of Lys-C and trypsin proteases. The resulting peptides were de-salted, dried by vacuum centrifugation and reconstituted in 0.1% trifluoroacetic acid containing indexed retention time (iRT) peptides (Biognosys, Schlieren, Switzerland).

Samples were analyzed on a QExactive HF mass spectrometer (Thermo Fisher Scientific) coupled with an Ultimate 3000 nano UPLC system (Thermo Fisher Scientific) and an EasySpray source (Thermo Fisher Scientific). Data were collected using data-independent acquisition (DIA), which fragments all peptides together and provides superior data for quantitation. Tryptic digests were spiked with iRT standards (Biognosys) and separated by reverse phase-HPLC on a nanocapillary column, 75 μm (internal diameter)× 50 cm 2 μm PepMap RSLC C18 column (Thermo Fisher Scientific) at 50°C. The mobile phase A consisted of 0.1% formic acid and the mobile phase B consisted of 0.1% formic acid/acetonitrile. Peptides were eluted into the mass spectrometer at 210 nl/min with each RP-LC run comprising a 125-min gradient from 1 to 5% B in 15 min, 5-45% B in 140 min. The raw files for DIA were collected using the following settings: one full MS scan at 120,000 resolution and a scan range of 300-1650 m/z with an automatic gain control (AGC) target of 3×10^6^ and a maximum injection time of 60 ms. This was followed by 22 (DIA) isolation windows with varying sizes at 30,000 resolution and an AGC target of 3×10^6^. The default charge state was 4, the first mass was fixed at 200 m/z, and the normalized collision energy for each window was stepped at 25.5%, 27% and 30%.

The suitability and sensitivity of the Q Exactive HF instrument was monitored by spiking in iRT peptides, followed by monitoring with the QuiC software (Biognosys). As a measure for quality control, we injected standard *Escherichia coli* protein digest between samples (one injection after every six biological samples) and collected the data in the data-dependent acquisition (DDA) mode, which enables measurement of the most abundant peptides in each cycle. The collected DDA data were analyzed in MaxQuant (Tyanova et al., 2016b) and the output was subsequently visualized using the PTXQC package (Bielow et al., 2016) to track the quality of the instrumentation.

The raw files for DIA analysis were processed with Spectronaut (Bruderer et al., 2015) version 16 in DirectDIA mode using reference *Mus muculus* proteome from UniProt (25,125 reviewed canonical and isoform proteins). The default settings in Spectronaut were used for peptide and protein quantification. Perseus (v1.6.14.0) (Tyanova et al., 2016a) was employed for data processing and statistical analysis using the MS2 intensity values generated by Spectronaut. Protein groups containing matches to contaminants were discarded. The data were log_2_ transformed and normalized by subtracting the median for each sample. The proteins with less than three valid values in at least one genotype were filtered out. A two-tailed unpaired Student's *t*-test was employed to identify differentially expressed proteins, and false discovery rate (FDR) was controlled with permutation-based methods. An adjusted *P*-value <0.05 was used as the significance threshold. Volcano plots were generated to visualize the affected proteins while comparing different groups of samples. The proteins that were exclusively detected in one experimental group were also reported for further bioinformatics analysis. The biological enrichment analysis was performed using the ToppGene Suite, with FDR controlled using the Benjamini–Hochberg procedure (Chen et al., 2009b). The mass spectrometry proteomics data have been deposited to the ProteomeXchange Consortium via the PRIDE (Perez-Riverol et al., 2022) partner repository with the dataset identifier PXD043613.

### Lipidomics and metabolomics

Metabolites were extracted from isolated RPE cells using 80 µl extraction buffer (40% acetonitrile/40% methanol/20% H_2_O containing 1 mM N-ethylmaleimide) on ice (Lu et al., 2018; Jankowski and Rabinowitz, 2022). Samples were centrifuged at 21,000 ***g***, 4°C for 20 min. Supernatants were collected and run by hydrophilic interaction chromatography (HILIC)-LCMS on a QExactive Orbitrap (Thermo Fisher Scientific) using the ion-switching method as previously described (Jankowski and Rabinowitz, 2022; Lu et al., 2018).

The water-insoluble pellet was re-suspended in 600 µl 50% methanol:50% H_2_O containing 0.1 N HCl and extracted further with 300 µl CHCl_3_. Samples were centrifuged at 21,000 ***g***, 4°C for 5 min. The CHCl_3_ fraction was collected using a glass syringe and transferred to a fresh glass tube. The acid fraction and insoluble protein from each sample were re-extracted with a fresh 300 µl portion of CHCl_3_. Samples were centrifuged again at 21,000 ***g***, 4°C for 5 min. The second CHCl_3_ fraction was collected and added to the first, and the combined CHCl_3_ extracts were dried under nitrogen gas (N_2_). Samples were re-suspended in 100 µl of 1:1:1 methanol:acetonitrile:isopropyl alcohol and run by reversed-phase LCMS on a QExactive Orbitrap using an ion-switching method as previously described (Papazyan et al., 2016). Cholesterol was detected as the [M+H–H_2_O]^+^ fragment (Gardner et al., 2017). LCMS data were analyzed using EL-MAVEN (Agrawal et al., 2019). Data visualization and statistical analysis were performed using GraphPad Prism v9. Statistical significance was assessed using two-tailed unpaired *t*-tests, followed by FDR correction using the two-stage step-up method of Benjamini, Krieger and Yekutieli with a cut-off of q=0.05.

Protein content was used for LCMS data normalization. The remaining insoluble pellet in 50% methanol:50% H_2_O containing 0.1 N HCl was spun down and the supernatant removed. Protein was extracted using 0.1 N NaOH at 37°C for 24 h and quantitated using the Pierce BCA assay.

### Statistical analysis

Unless otherwise described, the mean±s.e.m. was calculated for every group, with subsequent analysis done using Student's two-group, two-tailed unpaired *t*-test. For multiple comparisons, one-way ANOVA with post hoc pairwise comparisons using Dunnett's T3 multiple comparisons test was used. Statistical analyses were performed with GraphPad Prism v9.

## Supplementary Material

10.1242/dmm.050066_sup1 Supplementary information
