# Supplementary material for: Oxidative stress induces lysosomal membrane permeabilization and ceramide accumulation in retinal pigment epithelial cells
Source: Dis Model Mech. 2023 Jul 25;16(7):dmm050066. doi: 10.1242/dmm.050066 (PMC10399446; doi:10.1242/dmm.050066)
Supplement: Supplementary information [file dmm-16-050066-s1.pdf]

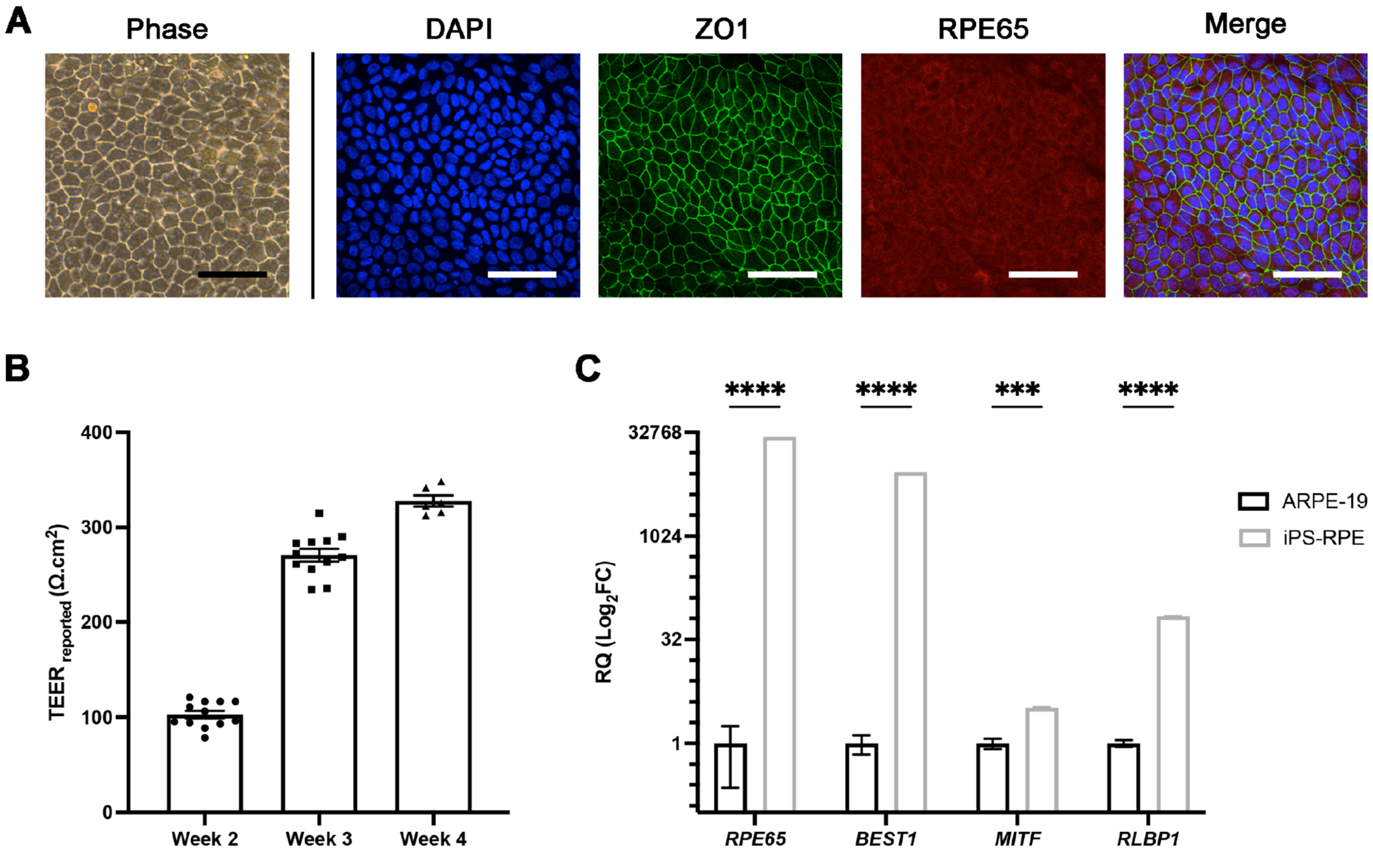

Fig. S1. Characterizationof iPS-RPE cells. (A) Representative images of mature iPSRPE cells under phase contrast and immunostained with Z01 (green) and RPE65 (red). Scale bar is 10 µm. Nuclei are labeled with DAPI (blue). **(B)** Transepithelial electrical resistance (TEER) of mature iPS-RPE cells grown on transwells. **(C)** Gene expression of mature RPE markers in iPS-RPE cells cultured for 4 weeks compared to ARPE-19 from n=3 replicates per cell line. Graphs represent mean values ± SEM. ***$p < 0.001$, ****$p < 0.0001$.

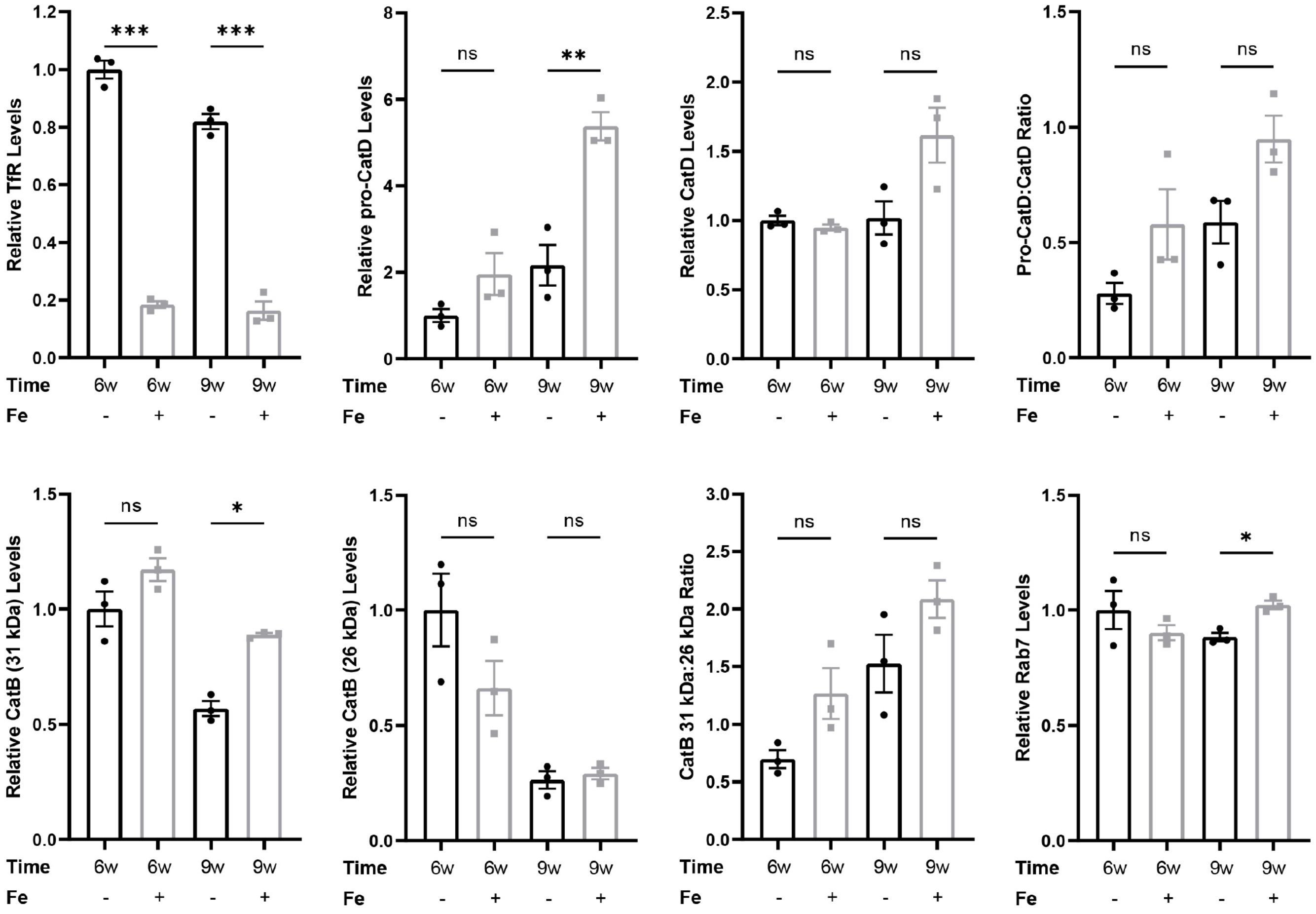

Fig. S2. Quantification of Western blots in iron-loaded iPS-RPE. Western blot images are shown in Fig. 1A. Graphs represent mean values ± SEM. *p < 0.05, **p < 0.01, ***p < 0.001.

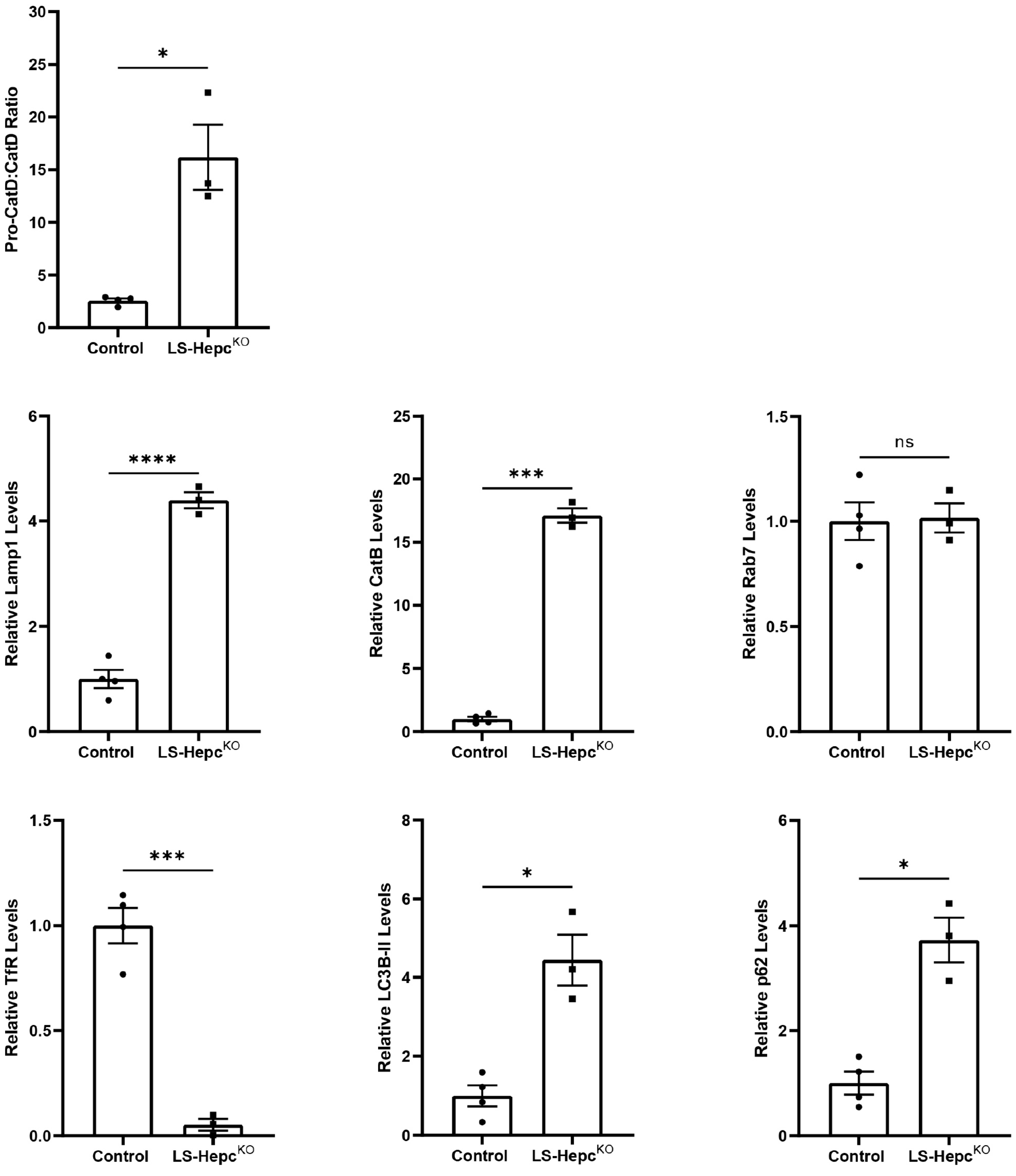

Fig. S3. Quantification of Western blots in 12-month LS-Hepc^{KO} mice. Western blot images are shown in Fig. 20. Graphs represent mean values ± SEM. *$p < 0.05$, ***$p < 0.001$, ****$p < 0.0001$. Control mice had genotype AlbCre-, Hepc$^{f/f}$.

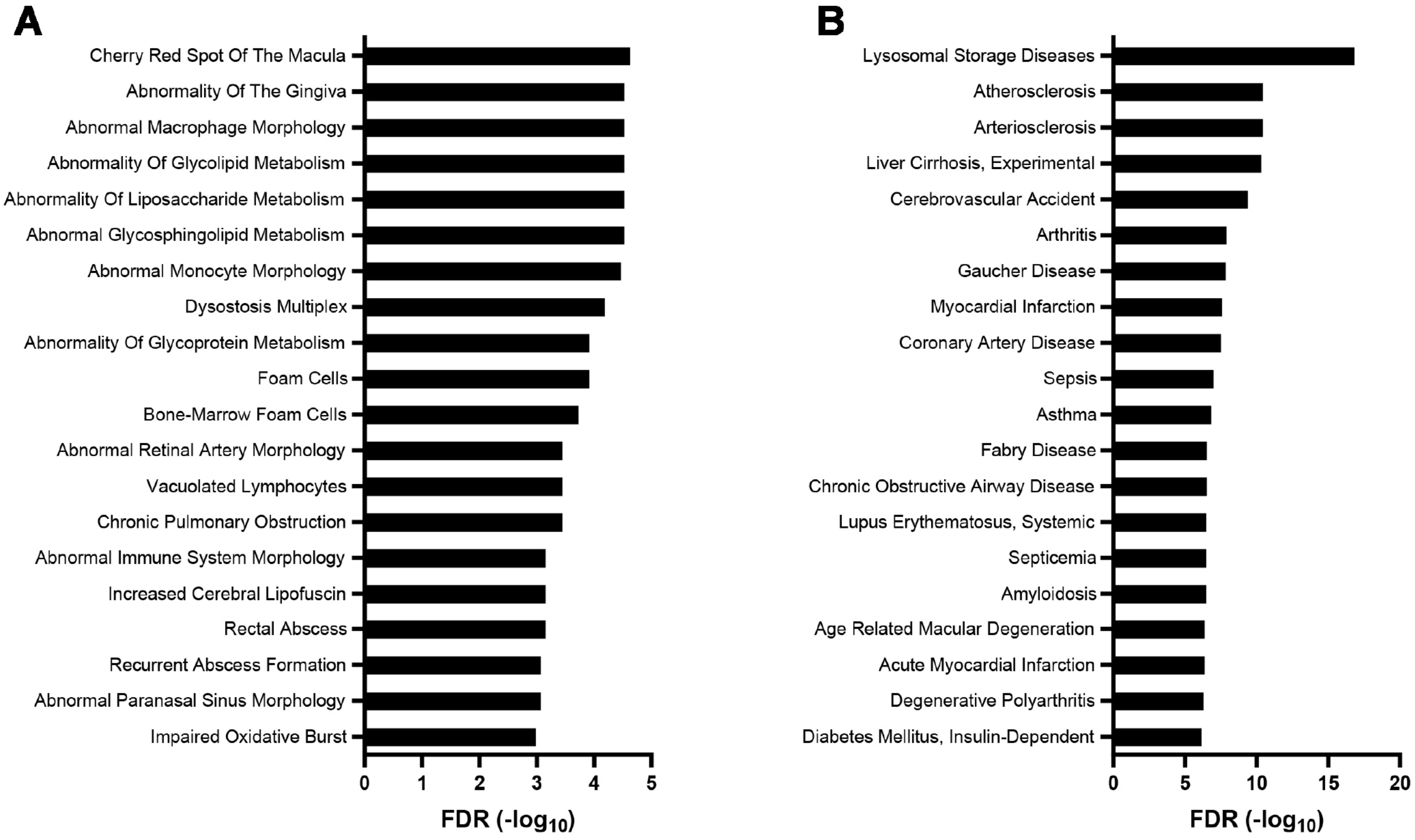

Fig. S4. Additional proteomics enrichment analysis on LS-Hepc^{KO} RPE. Enrichment analysis with the ToppGene Suite on 12-month LS-Hepc^{KO} vs control (AlbCre^{-}, Hepc$^{f/f}$) RPE for **(A)** human phenotype and **(B)** disease association. Disease associations in B are sourced from the DisGeNET BeFree system.

Disease Models & Mechanisms • Supplementary information

Table S1. Primary antibodies used for Western blot and immunofluorescence. Antibodies used for immunofluorescence are designated by IF, while those used for Western blot are designated by WB.

Antibody	Source	Identifier	Dilution
Mouse anti-CEP	Gift of John Crabb, Cleveland Clinic	N/A	1:200 (IF)
Mouse anti-methylglyoxal	Novus Biologicals	NBP2-59368	1:50 (IF)
Mouse anti-RPE65	ThermoFisher	#MA1-16578	1:100 (IF)
Rabbit anti-4-hydroxynonenal	Abcam	ab46545	1:100 (IF)
Rabbit anti-cathepsin B	Cell Signaling	D1C7Y	1:1000 (WB)
Rabbit anti-cathepsin D	GeneTex	GTX19555	1:1000 (WB), 1:100 (IF)
Rabbit anti-galectin-3	ThermoFisher	PA5-34819	1:100 (IF)
Rabbit anti-LC3B	Cell Signaling	#2775	1:1000 (WB)
Rabbit anti-light ferritin	Gift of M. Poli and P. Arosio, University of Brescia, Italy	E17	1:2500 (IF)
Rabbit anti-Rab7	Cell Signaling	D95F2	1:1000 (WB)
Rabbit anti-SQSTM1	Abcam	ab109012	1:5000 (WB)
Rabbit anti-transferrin receptor	ThermoFisher	PA5-27739	1:1000 (WB)
Rabbit anti-ZO1	ThermoFisher	#61-7300	1:100 (IF)
Rat anti-Iba1	Abcam	ab283346	1:100 (IF)
Rat anti-Lamp1	Developmental Studies Hybridoma Bank	1D4B	1:135 (WB), 1:100 (IF)
Rabbit IgG HRP Linked Whole Ab	Cytiva	NA934	1:5000 (WB)
ECL Rat IgG, HRP Linked	Cytiva	NA935	1:5000 (WB)
Donkey anti-Rat Cy3	Jackson ImmunoResearch Laboratories	712-165-153	1:200 (IF)
Donkey anti-Rat Alexa Fluor 488	Jackson ImmunoResearch Laboratories	712-545-153	1:200 (IF)
Donkey anti-Rabbit Alexa Fluor 488	Jackson ImmunoResearch Laboratories	711-545-152	1:200 (IF)
Donkey anti-Mouse Alexa Fluor 488	Jackson ImmunoResearch Laboratories	715-545-150	1:200 (IF)
Goat anti-Mouse Alexa Fluor 594	ThermoFisher	A-11020	1:500 (IF)
Goat anti-Rabbit Alexa Fluor 488	ThermoFisher	A-11034	1:500 (IF)

Table S2. Proteomics on RPE from 12-month-old LS-Hepc^{KO} RPE and age-matched controls

Click here to download Table S2

Table S3. Lipidomics in RPE from 12-month-old LS-Hepc^{KO} RPE and age-matched controls

Click here to download Table S3

Table S4. Metabolomics in RPE from 12-month-old LS-Hepc^{KO} RPE and age-matched controls

Click here to download Table S4

Disease Models & Mechanisms • Supplementary information
